# Integrated Computational Analysis Reveals Early Genetic and Epigenetic AML Susceptibility Biomarkers in Benzene-Exposed Workers

**DOI:** 10.3390/ijms26031138

**Published:** 2025-01-28

**Authors:** Silvia Vivarelli, Cigdem Sevim, Federica Giambò, Concettina Fenga

**Affiliations:** 1 Department of Biomedical and Dental Sciences, Morphological and Functional Imaging, Section of Occupational Medicine, University of Messina, 98124 Messina, Italy; federicagiambo@gmail.com (F.G.); cfenga@unime.it (C.F.); 2 Department of Medical Pharmacology, Faculty of Medicine, Kastamonu University, Kastamonu 37150, Turkey; cigdemsevim@kastamonu.edu.tr

**Keywords:** benzene exposure, occupational biomarkers, acute myeloid leukemia, computational study, gene expression, DNA methylation, miRNA regulatory network, risk assessment, occupational health

## Abstract

Benzene, a well-known carcinogenic airborne pollutant, poses significant health risks, particularly in industries such as petroleum, shoemaking, and painting. Despite strict regulations, chronic occupational exposure persists, contributing to the onset of acute myeloid leukemia (AML) and other malignancies. Benzene’s carcinogenicity stems from its metabolic activation, leading to increased oxidative stress, DNA damage, and cancer transformation. While its toxicity is well-documented, the link between genetic and epigenetic alterations and cancer susceptibility in exposed workers remains underexplored. This study aims to identify early biomarkers of benzene exposure and AML risk by analyzing gene expression and DNA methylation datasets from GEO DataSets, integrated with molecular pathway analyses, as well as miRNA-target and protein-protein network evaluations. This multi-approach led to the identification of nine deregulated genes (CRK, CXCR6, GSPT1, KPNA1, MECP2, MELTF, NFKB1, TBC1D7, ZNF331) in workers exposed to benzene, with NFKB1 showing strong discriminatory potential. Also, dose-dependent DNA methylation changes were observed in CXCR6 and MELTF, while selected miRNAs such as let-7d-5p, miR-126-3p, and miR-361-5p emerged as key post-transcriptional regulators. Furthermore, functional enrichment linked these genes to immune response, inflammation, cell proliferation, and apoptosis pathways. While network analyses highlighted NFKB1, CRK, and CXCR6 as central to benzene-associated leukemogenesis. Altogether, these findings provide novel insights into an early biomarker fingerprint for benzene exposure and AML susceptibility, supporting the future development of biomolecular-based targeted occupational health monitoring and personalized preventive strategies for at-risk workers.

## 1. Introduction

Benzene, a well-established carcinogenic airborne pollutant, represents a significant occupational health risk, particularly in industries such as petroleum refining, painting, shoemaking, and chemical manufacturing [1]. Benzene’s ubiquity extends beyond occupational settings, with environmental exposure contributing to public health concerns globally [2].

Despite regulatory efforts to limit exposure, workers in several sectors are still at risk of chronic low-dose exposure, which is immunosuppressive, causes hematotoxicity, and is strongly linked to the development of hematological malignancies [3]. In fact, the International Agency for Research on Cancer (IARC) has classified benzene as a Group 1 carcinogen, highlighting its established association with blood cancers, particularly adult acute myeloid leukemia (AML) [4]. The recent reduction of the occupational exposure limits within the European Union to 0.2 ppm highlights the growing concern surrounding benzene’s harmful effects, as well as the urgent need for the achievement of novel effective monitoring strategies for workers [5].

The carcinogenic effects of benzene are primarily attributed to its metabolic activation into reactive products, such as benzene oxide and hydroquinone, which induce oxidative DNA damage, chromosomal aberrations, and alterations in cellular signaling pathways [6]. Notably, benzene-induced oxidative stress can activate the p53 tumor suppressor pathway, critical for DNA damage repair and apoptosis, while also affecting key hematopoietic signaling pathways such as Hedgehog, Notch/Delta, and NF-κB. Once disrupted, these pathways contribute to leukemogenesis by triggering both inflammation and immune evasion [7]. Additionally, reactive metabolites of benzene activate genes primarily associated with the MAPK pathway (such as Foxo1 and Fgf3), which play a crucial role in benzene-induced hematotoxicity [8]. While the genotoxic consequences of benzene are well-documented, the precise molecular mechanisms by which it influences gene expression, particularly in workers chronically exposed to the substance, remain poorly explored [9].

Gene expression profiling, which reflects cellular responses to environmental stressors, provides a critical opportunity to identify how exposure to benzene affects gene regulation and to pinpoint pathways that may lead to malignancy [10]. Additionally, recent studies have suggested that epigenetic mechanisms, such as DNA methylation and miRNA regulation, play a key role in the development of leukemia following benzene exposure [11,12].

The goal is to identify early molecular alterations associated with occupational exposure by a computational approach that analyzes gene expression and DNA methylation data from both workers exposed to benzene and patients with AML [13]. Also, by performing network-based analyses, it will be examined how differentially expressed genes may interact within cellular pathways involved in immune response, inflammation, cell proliferation, metabolism, and programmed death. Finally, the combination of omics data, along with miRNA-target and protein-protein interaction analyses, will provide a comprehensive understanding of the underlying modulatory pathways and molecular players involved.

Overall, this combined approach could represent a crucial step for shedding light on novel genetic and epigenetic candidates for early detection of susceptible workers to improve preventive measures and ensure safer environments for subjects occupationally exposed to benzene.

## 2. Results

### 2.1. Differential Expression Analyses Disclose a Panel of Deregulated Genes in Benzene-Exposed Workers

**Figure 1:**
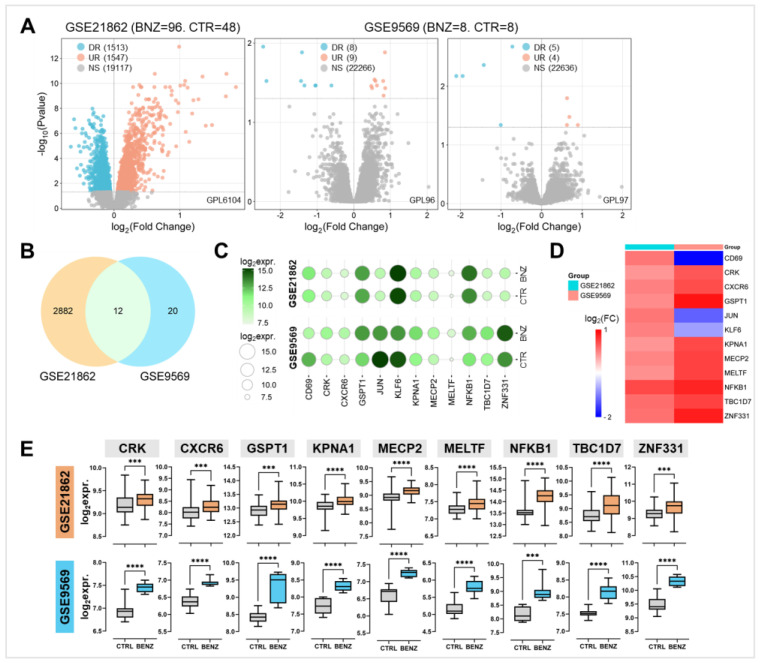
Differential expression analyses in the two GEO DataSets. (**A**) Volcano plots representing transcriptional expression data from GSE21862 (**left**) and GSE9569 (**right**). Light blue dots represent genes significantly downregulated (DR) in benzene-exposed versus non-exposed workers, while orange dots represent genes significantly upregulated (UR) in benzene-exposed versus non-exposed workers. Grey dots represent genes with non-significant (NS) changes. (**B**) Venn diagram illustrating the overlap of genes unique to each dataset and those shared between the datasets. (**C**) The bubble plot displays the relative expression of 12 significantly deregulated genes, with the size and color of the circles indicating the mean log_2_ expression values (log_2_expr.). (**D**) Heatmap of the averaged log_2_ fold change (log_2_FC) for each of the 12 genes across the two datasets. (**E**) Box plots showing the median expression levels ± SD of the 9 genes consistently upregulated in both datasets. *** *p* < 0.001; **** *p* < 0.0001.

Two gene expression datasets, GSE21862 and GSE9569, were analyzed to compare gene expression profiles between benzene-exposed workers and non-exposed controls. The volcano plots in Figure 1A illustrate the upregulated (UR) and downregulated (DR) genes in each dataset, identified by an adjusted *p*-value < 0.05. Among these, a total of 2894 genes in GSE21862 and 32 genes in GSE9569 were significantly UR or DR in chronically benzene-exposed workers compared to non-exposed individuals working indoors in office environments. Notably, 12 genes were commonly deregulated across both datasets: CD69, CRK, CXCR6, GSPT1, JUN, KLF6, KPNA1, MECP2, MELTF, NFKB1, TBC1D7, and ZNF331 (Figure 1B, Table S1).

The bubble plot in Figure 1C shows the average expression levels of these 12 genes. Analysis of the log_2_ fold changes (log_2_FC) for benzene-exposed versus non-exposed individuals revealed that nine of these genes—CRK, CXCR6, GSPT1, KPNA1, MECP2, MELTF, NFKB1, TBC1D7, and ZNF331—were consistently UR in both datasets, demonstrating a concordant pattern of deregulation (Figure 1D,E). These findings suggest that the deregulation of the nine key genes is a reliable hallmark of benzene exposure.

**Figure 2:**
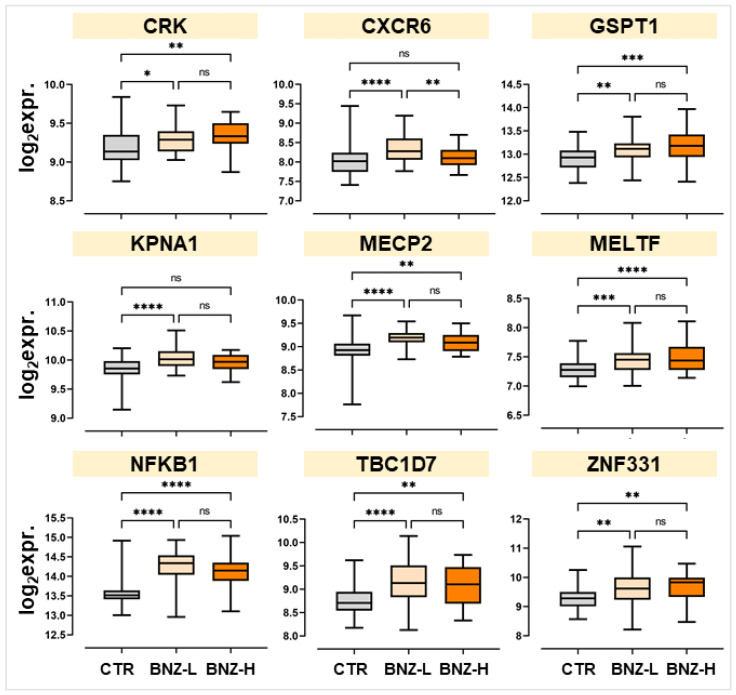
Stratification of significantly deregulated genes in GSE21862 based on benzene exposure. Box plots showing the median expression levels ± SD of the 9 benzene-related genes. Workers were stratified into low-dose benzene exposure (BZN-L: 0.1–5 ppm), high-dose benzene exposure (BZN-H: >5 ppm, including exposures exceeding 10 ppm), and non-exposed controls (CTR). * *p* < 0.05; ** *p* < 0.01; *** *p* < 0.001; **** *p* < 0.0001; ns = not significant.

In the GSE21862 dataset, benzene-exposed workers were stratified based on their daily occupational exposure levels into low-dose exposure (BZN-L), ranging from 0.1 to 5 ppm, and high-dose exposure (BZN-H), encompassing exposures of 5 ppm and above, including levels exceeding 10 ppm. As shown in Figure 2, the expression levels of KPNA1 and CXCR6 were significantly higher in the BZN-L group compared to the non-exposed controls (CTR). However, no significant differences were observed for these genes between the BZN-H group and CTR. Notably, the expression levels of CXCR6 were significantly lower in BZN-H compared to BZN-L.

For the remaining 7 genes (CRK, GSPT1, MECP2, MELTF, NFKB1, TBC1D7, and ZNF331), significant UR was observed in both BZN-L and BZN-H samples compared to CTR. However, there were no significant differences between the two exposure groups, indicating that higher exposure doses did not further alter the expression levels of these genes. These results highlight differential sensitivity among the genes to benzene exposure levels.

### 2.2. Correlation and ROC Analyses Reveal Gene Interaction Changes and Predictive Roles in Benzene-Exposed Workers

**Figure 3:**
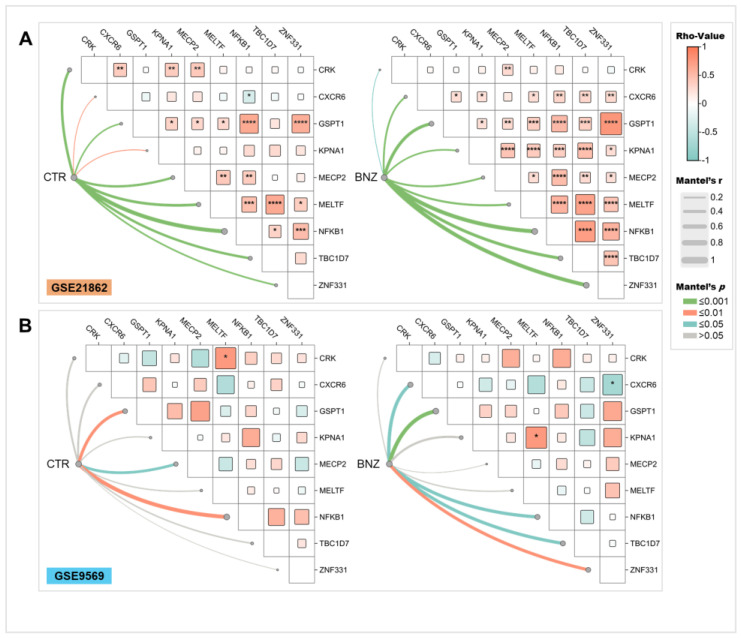
Correlation of the expression of significantly deregulated genes. Correlograms of Spearman correlations and Mantel analysis between deregulated benzene-related genes. (**A**) In the GSE21862 dataset, comparing CTR (control, **left**) and BNZ (benzene-exposed, **right**) samples. (**B**) In the GSE9569 dataset, comparing CTR (**left**) and BNZ (**right**) samples. Light red color within cells indicates positive correlations (rho values between 0 and 1), while light blue color indicates negative correlations (rho values between 0 and −1). The size of the squares in each cell is proportional to the magnitude of the rho value. Only significant correlations with *p*-values < 0.05 are reported. The lines denote Mantel test results, with the line width representing Mantel’s r statistic and the color representing Mantel significance (Mantel’s p). * *p* < 0.05; ** *p* < 0.01; *** *p* < 0.001; and **** *p* < 0.0001.

As shown in Figure 3, correlation and Mantel analyses revealed significant alterations in the association dynamics among the nine key genes in benzene-exposed (BZN) and non-exposed control (CTR) workers.

In particular, in GSE21862 (Figure 3A), CTR samples exhibited significant positive correlations among CRK, CXCR6, KPNA1, and MECP2, while CXCR6 negatively correlated with NFKB1. Positive correlations were also observed between GSPT1 and KPNA1, MECP2, MELTF, NFKB1, and ZNF331, with additional positive associations between MECP2, MELTF, and NFKB1, and between NFKB1, TBC1D7, and ZNF331. In BZN samples, the NFKB1-CXCR6 correlation shifted from negative to positive. While strengthened correlations were particularly evident for ZNF331 and GSPT1, those involving CRK, MECP2, and MELTF weakened in the exposed group (Table S2).

In GSE9569 (Figure 3B), fewer significant correlations were detected, likely due to the smaller sample size. In CTR samples, only a positive correlation between CRK and MELTF was significant. In BZN samples, a negative correlation emerged between CXCR6 and ZNF331, while a positive correlation appeared between MELTF and KPNA1. Consistent with GSE21862, the strength of selected correlations increased in BZN samples, particularly for CXCR6, GSPT1, TBC1D7, and ZNF331, as confirmed by the Mantel test (Figure 3B and Table S2). Interestingly, NFKB1 exhibited strong and significant strength in both the CTR and BZN groups across datasets, although slightly higher in non-exposed groups (Mantel r equal to 0.811, 0.767, 0.659, and 0.531, respectively, in CTR and BNZ of GSE21862 and GSE9569). The observed correlations highlight benzene exposure as a driver of distinct gene interaction patterns, with a selected strengthening of key correlations in both datasets, within exposed workers (i.e., GSPT1 and ZNF331), as well as non-exposed ones (i.e., MECP2, MELTF, and NFKB1).

Further support for the central role of NFKB1 in benzene exposure was provided by analysis of GSE119533 (Figure S1), which utilized the nCounter platform to validate the expression of a selection of 30 mRNAs across 59 occupationally benzene-exposed subjects and 37 non-exposed controls. NFKB1 was the only significantly upregulated gene in common with both GSE21862 and GSE9569, with an F value of 20.3 (FDR = 5.99 × 10^−10^, Figure S1A–C). Further stratification into different exposure groups revealed significant overexpression in CTR vs. B1 (benzene < 0.1 ppm) and B2 (benzene 5–10 ppm), but not in B3 (benzene > 10 ppm), indicating a nuanced exposure-response relationship (Figure S1D,E). Overall, NFKB1 exhibited a log_2_FC of 0.892 (FDR = 1.44 × 10^−11^) and demonstrated strong discriminatory power in the ROC analysis (AUC = 0.8552, *p* < 0.0001) (Figure S1F). These findings further underscore NFKB1 as a robust biomarker of benzene exposure, aligning with its critical role in the altered gene correlation networks observed.

**Figure 4:**
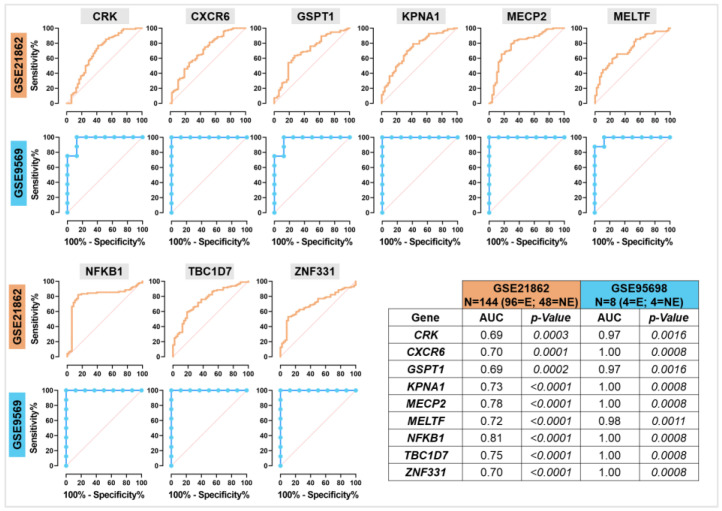
ROC curve analysis to assess the validity of gene expression in discriminating between benzene-exposed and non-exposed worker samples. ROC plots for the GSE21862 (light blue) and GSE9569 (orange) datasets are shown, with a corresponding table displaying the AUC and *p*-value for each of the nine benzene-associated genes. The AUC values reflect the discriminatory power of the genes in distinguishing exposed and non-exposed workers, with a higher AUC indicating stronger predictive capability.

Additionally, ROC curve analyses have been carried out to evaluate the ability of these 9 benzene-related genes to distinguish between BZN and CTR samples (Figure 4).

In GSE21862, AUC values ranged from 0.69 to 0.81, with NFKB1 demonstrating the highest discriminatory power (AUC = 0.81, *p* < 0.0001). In GSE9569, due to the limited number of cases, all genes showed exceptionally high AUC values (≥0.97), achieving perfect discrimination for several genes. Altogether, these findings highlight the significance of correlation dynamics and the predictive power of these nine genes as suggested biomarkers of benzene exposure. The high AUC values further validate their diagnostic power, with GSE9569 results likely benefiting from a smaller, more homogeneous cohort, while GSE21862 emphasizes their broader applicability across diverse populations.

### 2.3. Methylation Analysis Reveals Differential DNA Methylation Patterns in Benzene-Exposed Workers

**Figure 5:**
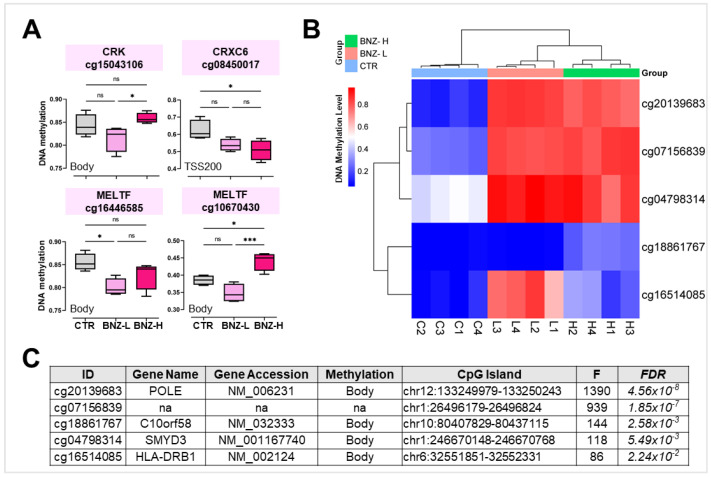
DNA methylation dataset analysis (GSE50967). (**A**) Box plots showing the median DNA methylation levels ± SD of CRK (cg15043106), CXCR6 (cg08450017), MELTF (cg16446585), and MELTF (cg10670430) in BNZ-L (low exposure to benzene: 0.06 ± 0.01 mg/m^3^, equivalent to 0.02 ppm), BNZ-H (high exposure to benzene: 7.68 ± 2.57 mg/m^3^, equivalent to 2.59 ppm), and CTR (non-exposed controls: 0.06 ± 0.01 mg/m^3^, equivalent to 0.02 ppm). (**B**) Heatmap of the mean DNA methylation levels of the five most deregulated DNA methylation hotspots, grouped into BNZ-L (L1-4), BNZ-H (H1-4), and CTR (C1-4). (**C**) Table reporting the main features of DNA methylation hotspots, including the gene ID, gene name, gene accession, methylation level, CpG island status, F-value, and FDR. * *p* < 0.05; *** *p* < 0.001; and ns = not significant.

Methylation data from the GSE50967 dataset were analyzed to investigate DNA methylation alterations in 9 key genes found deregulated with benzene exposure in workers compared to controls. As shown in Figure 5, for CRK, hypomethylation was observed in the body of the gene in the benzene low-exposed (BZN-L) group relative to controls, although this difference did not reach statistical significance. Interestingly, methylation levels of CRK were significantly restored in the benzene high-exposed (BZN-H) group compared to BZN-L, suggesting a potential dose-dependent regulation of methylation. In CXCR6, significant hypomethylation was detected at the TSS200 in the BZN-H group when compared to controls, indicating that benzene exposure may induce specific epigenetic changes at the transcriptional initiation site. Additionally, the methylation status of MELTF also showed significant variations: one probe in the gene body exhibited decreased methylation in BZN-L, while another probe demonstrated a significant increase in methylation in the BZN-H group relative to controls. This suggests a dose-dependent methylation response for MELTF. Overall, the methylation analysis highlighted distinct alterations in DNA methylation patterns for CRK, CXCR6, and MELTF, particularly in the BZN-H group, which exhibited more marked changes compared to both the BZN-L and control groups (Figure 5A).

As shown in Figure 5B,C, Further stratification of the dataset based on benzene exposure revealed significant methylation changes in several coding and non-coding genomic regions, including POLE, C10orf58, SMYD3, and HLA-DRB1, with FDR values ranging from 4.56 × 10^−8^ to 2.24 × 10^−2^.

### 2.4. Expression Patterns of Benzene-Associated Genes in AML Highlight Potential Links Between Occupational Exposure and Leukemogenesis

**Figure 6:**
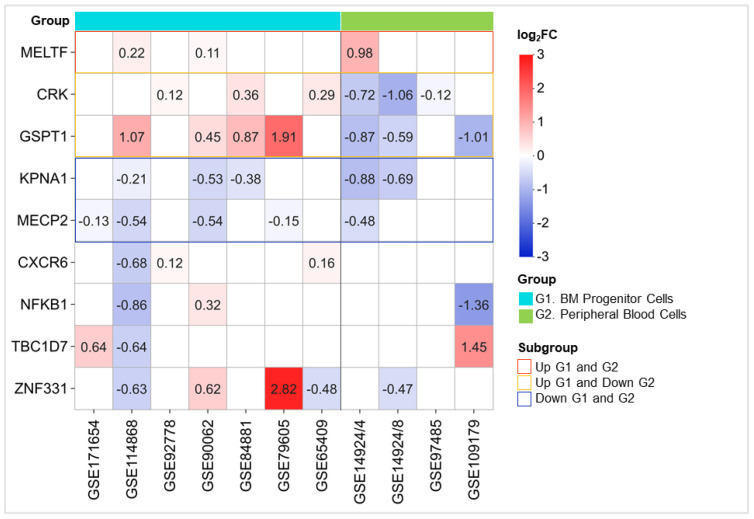
Gene expression matrix of 9 benzene-associated genes in AML GEO gene expression datasets. Boxes represent the averaged log_2_ fold change (log_2_FC) for each of the 9 genes. Red boxes indicate upregulated genes with log_2_FC between 0 and 3, while blue boxes represent downregulated genes with log_2_FC between 0 and −3. Unfilled white boxes indicate genes with non-significant fold changes. The datasets are grouped into G1 (light blue) with bone marrow (BM) progenitor cell-derived samples and G2 (light green) with peripheral blood nucleated cell-derived samples. MELTF is consistently upregulated in both G1 and G2 (red boxes), while CRK and GSPT1 show upregulation in G1 but downregulation in G2 (yellow boxes). KPNA1 and MECP2 are consistently downregulated in both G1 and G2 (blue boxes).

To explore the association between benzene exposure and leukemogenesis, benzene-associated gene expression profiles from nine datasets comparing AML patients (AML) to healthy controls (H) have been analyzed. Stratifying samples by origin (bone marrow vs. peripheral blood), distinct patterns of deregulation were observed (Figure 6).

In detail, MELTF consistently showed upregulation across all samples, irrespective of tissue type, highlighting its potential role as a broadly responsive marker in AML. Conversely, CRK and GSPT1 were upregulated in BM samples but downregulated in peripheral blood nucleated cells, suggesting a tissue-specific regulation influenced by the leukemic environment. Notably, KPNA1 and MECP2 exhibited consistent downregulation across both bone marrow and blood samples, indicating a uniform response to AML across different tissues, although opposite to the upregulation observed in benzene-exposed workers. The remaining genes displayed no consistent pattern of deregulation, emphasizing heterogeneity in their expression profiles across the datasets. Additionally, ROC analyses evidenced a significant discriminatory power of the five genes in distinguishing AML from healthy samples (Figure S2).

These findings partially contrast with benzene-exposed worker datasets, where KPNA1 and MECP2 were upregulated, indicating putative early stress-response roles that are lost during malignant transformation. Intriguingly, the consistent upregulation of MELTF in both AML and benzene exposure suggests its involvement in pathways linking environmental exposure to hematologic malignancies.

### 2.5. Functional and Network Analyses Highlight the Biological Roles and Interactions of the Benzene-Associated Gene Panel

**Figure 7:**
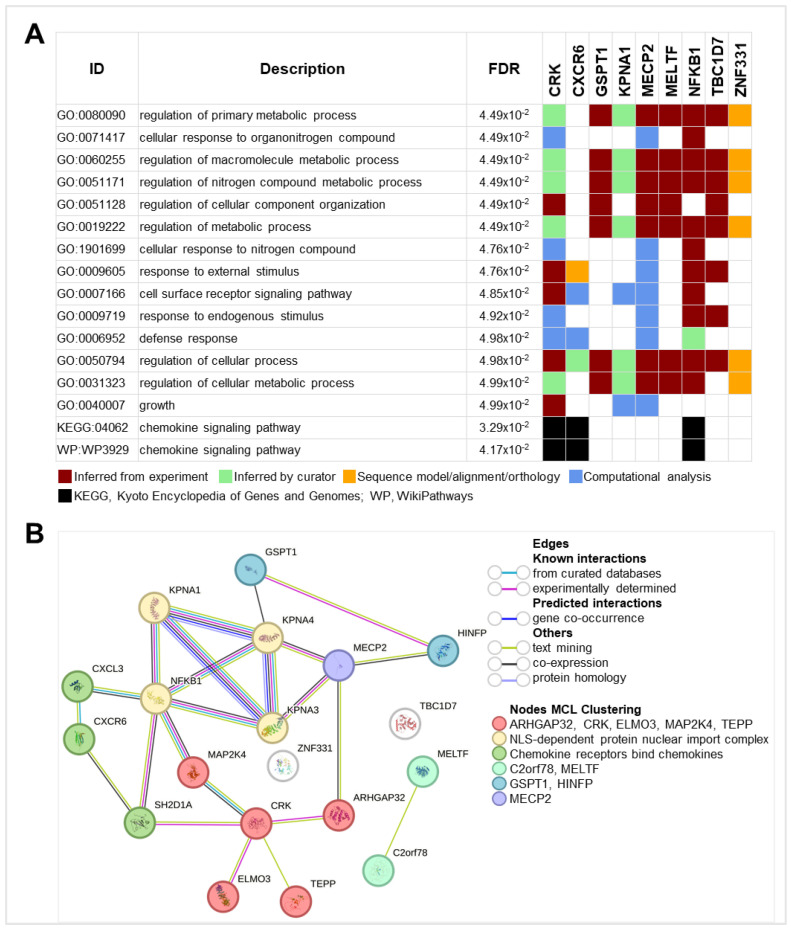
g:GOSt and STRING functional network analyses of the panel of nine differentially expressed benzene-related genes. (**A**) Table summarizing the multi-query results with significant FDR values for 14 Biological Process Gene Ontology (BP:GO) terms, 1 Kyoto Encyclopedia of Genes and Genomes (KEGG) pathway, and 1 WikiPathways (WP) pathway. Square colors indicate different types of evidence, as described in the bottom of the table. (**B**) STRING plot showing protein–protein associations, where edges represent interactions between proteins and nodes represent the query proteins and their first shell of interactors. The colors of the edges, as well as the node clusters, are indicated in the figure.

Functional enrichment analysis using g:GOst identified key biological processes (BPs) significantly associated with the nine-gene panel (Figure 7A). The regulation of primary metabolic processes emerged as a central feature, involving all genes except CXCR6, while the modulation of cellular processes included all nine genes, underscoring the panel’s broad influence on essential cellular functions. Interestingly, CXCR6 played a distinct role in response to external stimuli, cell surface receptor signaling, and defense responses, highlighting its involvement in immune and stress-related pathways. Additionally, CXCR6, CRK, and NFKB1 were implicated in the chemokine signaling pathway in both the KEGG and WikiPathways databases, pointing to their potential roles in immune modulation and inflammation.

Additionally, STRING network analysis (Figure 7B) revealed a dense web of functional interactions between the protein products of the genes, with the exception of ZNF331 and TBC1D7, which appeared functionally isolated. The analysis identified distinct clusters of interactions. One cluster suggested roles in cytoskeletal remodeling and intracellular signaling, highlighting genes such as CRK and MAP2K4, while another emphasized nuclear-cytoplasmic transport processes involving KPNA1 and NFKB1. A separate cluster underscored the chemokine signaling roles driven by CXCR6, further supporting its immune-related functions. Additional clusters hinted at roles in iron homeostasis, translational control, and chromatin dynamics, with MECP2 showing indirect interactions with both NFKB1 and GSPT1.

Table S3 highlights the significant networking interactions observed in STRING. Four of the nine genes (KPNA1, NFKB1, CRK, and CXCR6) are involved in several key processes. KPNA1, in particular, emerges as a pivotal player in nucleocytoplasmic transport, mediating the import of proteins bearing nuclear localization signals (NLS) through its association with key components such as KPNA3 and KPNA4. Also, the results underscore the significant involvement of NFKB1, CRK, and CXCR6 in immune-related pathways. NFKB1 is notably implicated in the TNF signaling pathway and the chemokine signaling pathway, both of which are crucial for inflammation and immune response modulation. The participation of these genes in the immune system is further supported by their association with cytokine signaling and the ISG15 antiviral mechanism. Moreover, KPNA1 and NFKB1 feature prominently in pathogen response pathways.

Together, these findings underscore the coordinated involvement of the nine-gene products panel in regulating subcellular, metabolic, immune, and inflammatory processes, highlighting their relevance in the cellular response to benzene exposure.

### 2.6. Interactome Analyses Highlight CRK, GSPT1, KPNA1, MECP2, and MELTF as Central Nodes in AML Pathogenesis with Distinct Prognostic Implications

**Figure 8:**
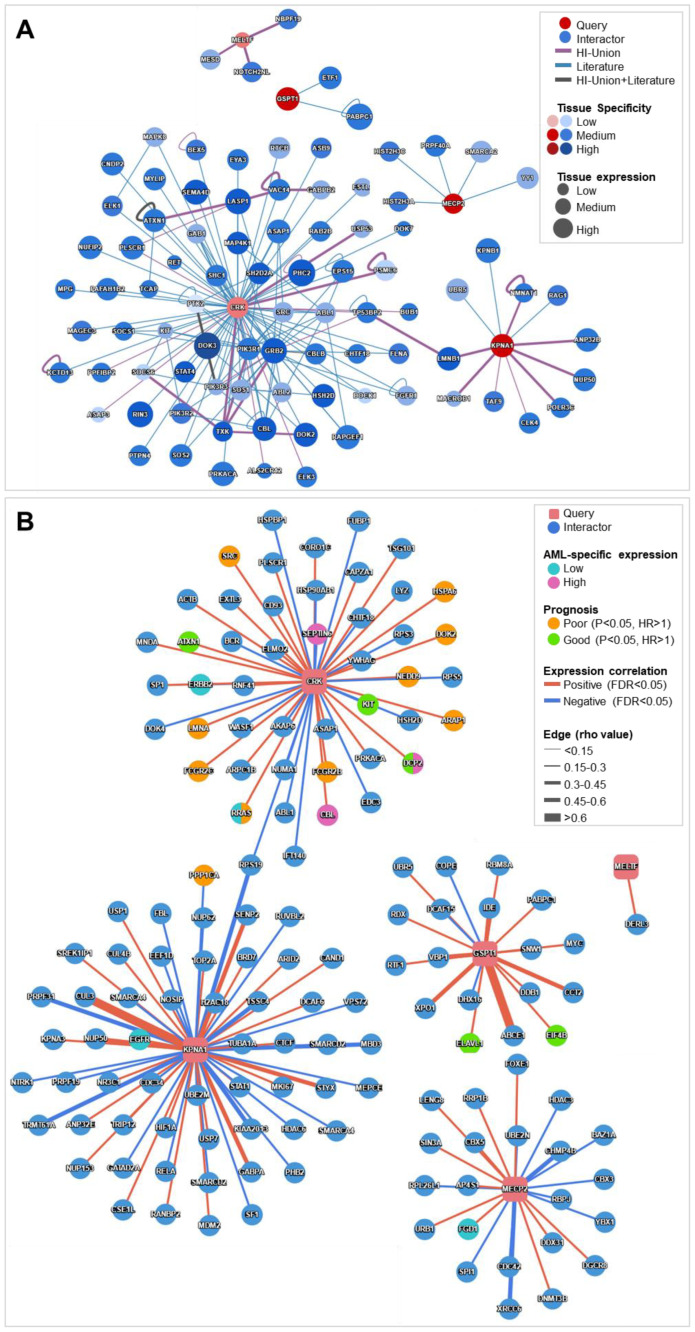
Protein-protein interaction networks for five benzene-associated genes consistently deregulated in AML samples. (**A**) Protein interaction network generated using the Human Reference Interactome (HuRI) in blood tissue. (**B**) Protein interaction network generated using the Protein Interaction Network Analysis (PINA 3.0) platform, in the context of AML (The Cancer Genome Atlas, TCGA dataset). Color codes for edges and nodes in both networks are specified within the figure (**upper left**).

The protein interaction networks were analyzed using the Human Reference Interactome (HuRI) and Protein Interaction Network Analysis (PINA 3.0) platforms to investigate the functional relationships occurring between the five benzene- and AML-associated genes within the context of AML pathogenesis. Figure 8A illustrates the protein interaction network generated using HuRI, focusing on protein–protein interactions within blood tissue.

This network revealed several significant interactions among the five differentially regulated genes in AML (CRK, GSPT1, MECP2, KPNA1, and MELTF). In particular, CRK emerged as the gene with the broadest interaction profile, engaging with a variety of proteins involved in cellular processes such as signaling and cell cycle regulation, including LASP1, MYLIP, SH2D2A, PRKACA, FGFR1, EPS15, PTPN4, and MAP4K1 (Table S4).

These interactions suggested that CRK plays a central role in modulating key cellular functions critical to cellular transformation. Intriguingly, both GSPT1 and KPNA1, which were highly expressed in healthy blood cells, showed notable interactions within this network. GSPT1 interacted with PABPC1 and ETF1, which are involved in protein synthesis and translation regulation. KPNA1 interacted with NUP50, KPNB1, and several other proteins related to nuclear transport and chromatin regulation. An important finding in the HuRI network was the indirect interaction between CRK and KPNA1, mediated through LMNB1. Additionally, MELTF interacted with NOTCH2NL and NBPF19. Finally, MECP2, on the other hand, interacted with the chromatin remodeling factors SMARCA2 and YY1.

Figure 8B shows the protein interaction network generated using the PINA 3.0 platform, focusing on protein–protein interactions in the context of AML (TCGA dataset). The analysis revealed that all protein products from the genes CRK, GSPT1, KPNA1, MECP2, and MELTF interacted with proteins that were highly expressed in this cancer type (Table S5). Remarkably, CRK interacted with SEPTIN6 (negative correlation) and CBL (positive correlation), both of which are involved in cell signaling and cytoskeletal regulation. Additionally, CRK interacted directly with proteins associated with both poor and favorable prognostic outcomes in AML. Specifically, CRK positively interacted with SRC, HSPA6, LMNA, and FCGR2B and C, which are linked to poor prognosis, while it negatively interacted with KIT and positively interacted with ATXN1, both of which are associated with a better prognosis. These interactions suggested that CRK played a pivotal role in modulating various cellular processes that influence AML progression.

Moreover, CRK was indirectly connected to KPNA1 via RPS19. GSPT1 also interacted positively with ELAVL1 and EIF4B, both of which are associated with favorable prognostic outcomes in AML. Furthermore, KPNA1 negatively interacted with PPP1CA, a protein linked to poor prognosis, suggesting a potential role of KPNA1 in modulating negative prognostic pathways in leukemia.

Overall, the interactome analyses identified CRK, GSPT1, KPNA1, MECP2, and MELTF as pivotal nodes in the blood compartment. Also, each of these gene products demonstrated distinct interactions relevant to leukemogenesis, suggesting their central role in AML pathology.

### 2.7. miRNet Analysis Highlights Key Tissue-Specific and AML-Specific miRNA-Target Regulatory Patterns

**Figure 9:**
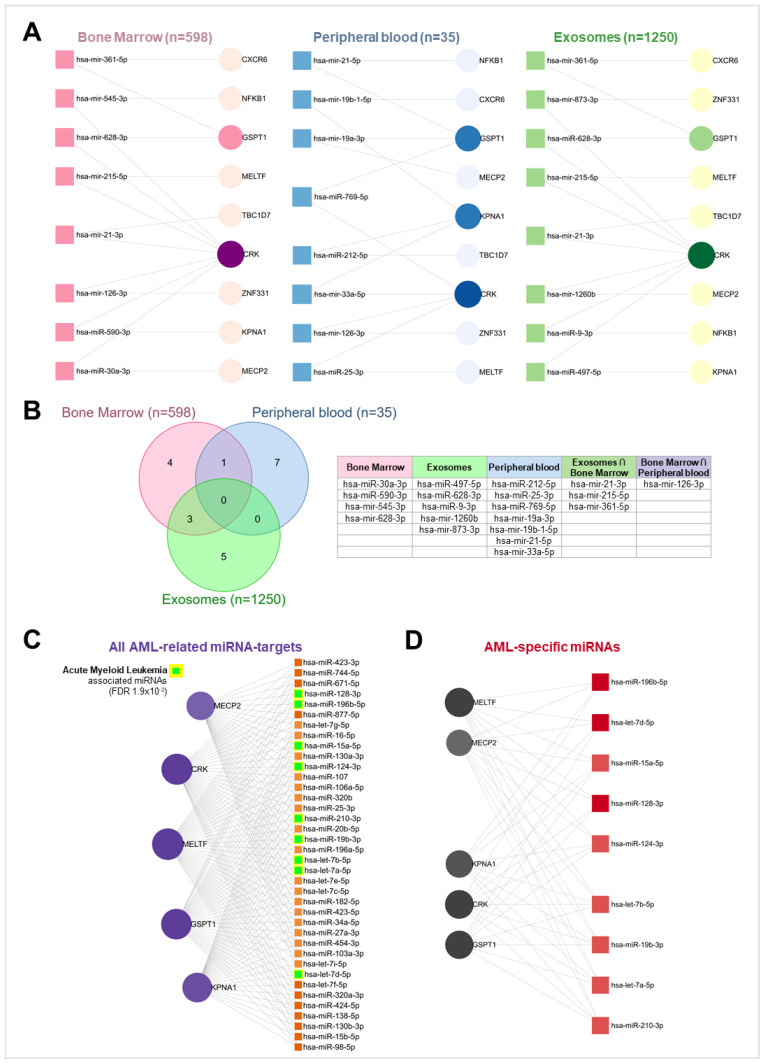
miRNet miRNA-target interaction network of differentially expressed benzene-related gene targets. (**A**) Network plots highlighting the interactions (grey nodes) between the 9 benzene-associated gene targets and 8 miRNAs in bone marrow (**left**), peripheral blood (**center**), and exosome (**right**) samples. (**B**) Venn diagram showing intersections between miRNet queries and a table listing miRNAs that are post-transcriptional regulators of benzene-related gene targets belonging to each Venn subgroup. (**C**) Network plot depicting the interactions (grey nodes) between 5 benzene- and AML-associated gene targets and 38 miRNAs; miRNAs specifically involved in AML pathology are highlighted in green and yellow. (**D**) Network plot of 5 benzene- and AML-associated gene targets and 9 miRNAs of the AML-disease subgroup, with interactions (grey nodes).

The miRNet analysis provided critical insights into the miRNA-target interaction networks across different tissues and diseases, particularly highlighting regulatory mechanisms in AML. Figure 9A,B illustrate the miRNA-target interaction networks for three distinct tissue types—bone marrow, peripheral blood, and exosomes—for all nine benzene-related genes. While most miRNAs were tissue-specific, several were shared between tissues.

Notably, hsa-miR-21-5p and hsa-miR-25-3p overlapped between exosomes and bone marrow, while bone marrow and peripheral blood shared hsa-miR-126-3p, hsa-miR-361-5p, and hsa-miR-21-5p. These findings underscored the tissue-specific roles of miRNAs in the post-transcriptional regulation of the benzene-target genes, with selected overlaps between bone marrow, peripheral blood, and exosomes suggesting key regulatory interactions in these areas.

Figure 9C shows the subset of miRNAs potentially regulating the post-transcriptional levels of the subgroup of five AML-related genes across all available samples within the miRNet database. Overall, a total of 38 miRNAs were identified as significantly interacting with these genes. Among these, two miRNAs belong to the let-7 family (hsa-let-7a-5p and hsa-let-7f-5p), and three to the miR-103 family (hsa-miR-103a-3p and hsa-miR-107).

The subsequent disease pathway analysis performed on these 38 miRNAs, summarized in Table S6, identified several significant disease subgroups and their associated pathologies. Among the most prominent subgroups were cancers, with a particular emphasis on leukemias, including AML, which may have been linked to environmental exposures such as chronic benzene exposure. Additionally, carcinomas, encompassing a wide range of epithelial cancers, as well as neoplasms, were identified. Other disease subgroups included cardiovascular diseases, metabolic disorders, and liver and kidney diseases, underscoring the broader impact of these miRNAs on various health conditions potentially associated with benzene exposure.

Specifically for AML, the pathway analysis linked nine key miRNAs as selected regulators of CRK, MELTF, MECP2, KPNA1, and GSPT1 targets: hsa-let-7b-5p and hsa-let-7a-5p (let-7 family); hsa-let-7d-5p and hsa-miR-124-3p (mir-124 family); hsa-miR-128-3p and hsa-miR-15a-5p (miR-15 family); hsa-miR-196b-5p, hsa-miR-19b-3p, and hsa-miR-210-3p (Figure 9C). This analysis underscored the possible role of these miRNAs as biomarkers for AML, highlighting their link with chronic benzene exposure.

## 3. Discussion

The use of a multi-omics approach to characterize the toxic effects of benzene on workers has been widely discussed for nearly two decades [14]. Despite a growing body of preclinical evidence, epidemiological data in worker populations remain limited [15]. Consequently, an actionable panel of deregulated genes for real-world applications has yet to be established. Although occupational exposure is tightly regulated, it persists as a significant concern in industrialized societies, posing risks to both workers and the general population [16]. Actually, chronic exposure to benzene, even at low levels, induces hematotoxicity, as a precursor to leukemogenesis, eventually progressing to AML and other leukemias or lymphomas [17].

Key inquiries remain: what do we know about the changes in gene expression profiles and DNA methylation patterns in benzene-exposed workers? Do any genes with altered transcriptional profiles in exposed individuals exhibit similar changes in patients with established AML? This study addresses these critical issues. To advance current knowledge and identify a transcriptional, epigenetic, and post-transcriptional signature of benzene susceptibility and exposure, it is essential to consolidate findings from different datasets, including those involving low-dose exposures. From a systems biology perspective, it is also vital to complement transcriptomic and epigenomic data with correlation and predictive analyses, as well as network and interactome studies [13].

A differential expression analysis comparing benzene-exposed workers and non-exposed controls was conducted using two publicly available gene expression datasets (GSE21862 and GSE9569). Of over 2000 deregulated genes, 12 were consistently deregulated across both datasets, with nine genes—CRK, CXCR6, GSPT1, KPNA1, MECP2, MELTF, NFKB1, TBC1D7, and ZNF331—showing consistent upregulation. This suggests a conserved molecular hallmark of benzene exposure in workers. Further analysis of GSE21862, stratifying workers by exposure levels, identified patterns of differential sensitivity. KPNA1 and CXCR6 were selectively upregulated in workers chronically exposed to low levels (0.1–5 ppm), whereas the other seven genes were upregulated regardless of exposure level (from 0.1 to over 10 ppm).

Previous gene expression analyses using U133 array technology on PBMC samples from shoe manufacturing workers exposed to high benzene levels corroborate several findings from this study. Notably, CRK and ZNF331 were consistently upregulated [18]. CRK, an oncogene involved in cellular transformation, is currently being investigated as a therapeutic target in leukemia [19,20]. ZNF331, a zinc-finger transcription factor, has been found often hypermethylated (a status linked to increased expression) within peripheral blood leukocytes in various cancers [21,22]. Additionally, CXCL16, a member of the CXC chemokine family, was previously identified as upregulated in benzene-exposed workers [18]. In our study, its receptor, CXCR6, was found to be upregulated. Indeed, the CXCL16/CXCR6 axis plays a crucial role in immune modulation and is particularly implicated in AML pathogenesis [23,24]. Consistent with our findings, the GSE119533 study, which used nCounter technology, confirmed NFKB1 as a robust biomarker for benzene exposure [25]. However, a study by Thomas et al. using RNA-seq on PBMC samples from 250 benzene-exposed workers and 140 controls failed to identify any of the genes from our panel as significantly deregulated. Only two ZNF factors from the same family as ZNF331 (ZNF703 and ZNF420) were upregulated in exposed workers [26]. This discrepancy may be attributed to the dissimilar technologies used (RNA-sequencing versus RNA microarray) [27].

Following correlation analyses, a higher number of positive correlations among the nine genes identified in our study were observed in benzene-exposed subjects. However, the pattern of Mantel correlation strengths varied: in benzene-exposed workers, the strength of correlations involving GSPT1 and ZNF331 increased, whereas in controls, correlations involving MECP2, MELTF, and NFKB1 were slightly stronger. This variation implies that benzene exposure may selectively enhance or decrease specific gene interactions, contributing to the disruption of normal cellular processes [4].

Intriguingly, ROC curve analyses of these nine genes in both datasets demonstrated their ability to distinguish between exposed and non-exposed workers, with NFKB1 showing the highest significant discriminatory power in GSE21862 (Figure 4). To the best of our knowledge, this is the first study to complement transcriptomic data with correlation and ROC analyses, underscoring the predictive power of the identified gene panel. The upregulation of NFKB1 upon benzene exposure is not surprising, and it is supported by growing evidence highlighting the role of NF-κB pathway activation in response to benzene metabolites, particularly hydroquinone, especially in circulating T cells [28,29]. This result emphasizes the significance of the NF-κB inflammatory pathway in targeted therapy, with possible translational implications for benzene toxicology risk assessment [30].

To further investigate DNA methylation alterations in the identified genes, the GSE50967 dataset was examined. CXCR6 exhibited significant hypomethylation at its transcriptional start site in the high-exposure group. In contrast, for MELTF, one probe within the gene body showed decreased methylation in the low-exposure group, while another showed increased methylation in the high-exposure group, indicating a dose-dependent response. The gene MELTF encodes for melanotransferrin, a protein involved in iron metabolism and the regulation of ferroptosis, which has been recently linked to benzene-induced hematotoxicity in animal models [31,32].

To elucidate the biological roles and interactions of the nine benzene-associated genes, functional enrichment and network analyses were performed. In detail, g:GOst analysis highlighted the regulation of primary metabolic and cellular processes, emphasizing the gene panel’s broad influence on essential biological functions. CXCR6 was found to be involved in immune and stress-related pathways, including cell surface receptor signaling and defense responses. Additionally, CXCR6, CRK, and NFKB1 were implicated in the chemokine signaling pathway, linking them to immune modulation and inflammation. Furthermore, STRING network analysis revealed a dense web of functional interactions among the gene products. For instance, CRK was primarily involved in cytoskeletal remodeling and intracellular signaling (in association with MAP2K4, ELMO3, and other gene products). While KPNA1 and NFKB1 played roles in nuclear-cytoplasmic transport. Finally, both CXCR6 and NFKB1 were involved in chemokine signaling. These observations align with previous studies demonstrating the central role of the immune system and inflammation in responding to oxidative stress induced by benzene poisoning [16,33].

Subsequent miRNet analysis identified several miRNAs regulating benzene-related genes across different tissues, with distinct patterns between bone marrow, peripheral blood, and exosomes. The miRNA-target interaction networks showed that most miRNAs were tissue-specific, although some were shared across tissues. Particularly, hsa-miR-21-5p and hsa-miR-25-3p overlapped between exosomes and bone marrow, while hsa-miR-126-3p, hsa-miR-361-5p, and hsa-miR-21-5p were shared between bone marrow and peripheral blood, suggesting their broad regulatory roles in response to benzene exposure. Consistent with our findings, miR-126-3p was found to be deregulated in hematopoietic progenitor cells treated with benzene, while plasma levels of miR-361-5p were significantly reduced in workers chronically exposed to high benzene levels [34,35]. Hence, it can be suggested that these miRNAs play a crucial role in the post-transcriptional regulation of benzene-related genes across different tissues in occupational settings.

To further assess the relationship between benzene exposure and leukemogenesis, gene expression profiles from nine AML datasets were analyzed, stratified by tissue type (bone marrow vs. peripheral blood). In both bone marrow and peripheral blood nucleated cells, MELTF was consistently upregulated, suggesting its potential role as a broad marker for AML. Accordingly, the MELTF gene codes for one of the first melanoma tumor antigens identified, and it is involved in multiple steps of tumor cellular transformation [36]. Although its role in AML is yet to be elucidated, MELTF represents a known negative prognostic marker in colon and gastric cancers [37,38]. Our analysis also revealed that the oncogenes CRK and GSPT1 were upregulated in bone marrow cells but downregulated in peripheral blood, indicating tissue-specific regulation influenced by the leukemic environment [39]. Interestingly, benzene oxide, a toxic metabolite of benzene, acts as a substrate for GSTP1, which has been linked to benzene-induced hematotoxicity [40,41]. Moreover, GSPT1, a key factor in translation termination, has recently been identified as an oncogenic driver and holds promise as a target for developing anti-AML drugs [42,43,44,45].

In addition, KPNA1 and MECP2 were consistently downregulated in both blood cell- and bone marrow-derived AML samples, contrasting with their upregulation observed in benzene-exposed workers expression datasets. This difference may reflect a shift in gene expression during malignant transformation; however, it appears in contrast with several findings. In detail, although KPNA1 (a nuclear import protein) is overexpressed in several cancers, it was found downregulated in AML bone marrow cells in this study, contradicting previous reports [46,47]. Similarly, MECP2, a transcriptional modulator that binds methylated DNA, primarily known for its role in Rett syndrome, is generally overexpressed in many cancers, although its role in leukemia remains underexplored [48,49].

ROC analyses confirmed the strong discriminatory power of MELTF, CRK, GSPT1, KPNA1, and MECP2 in distinguishing AML from healthy controls, underscoring their role as key biomarkers for AML susceptibility, especially in workers exposed to benzene. This finding suggests a new panel of genes that could serve as early biomarkers for AML in the context of benzene exposure, offering novel insights into occupational health surveillance.

In additional support of this perspective, protein-protein interaction network analyses using HuRI revealed CRK as the gene with the most extensive interaction profile, engaging with proteins such as LASP1, MYLIP, SH2D2A, and FGFR1, underscoring its central role in AML-related cellular processes [50,51]. Also, GSPT1 interacted with PABPC1 and ETF1, proteins involved in translation regulation [52]. While KPNA1 interacted with nuclear transport proteins such as NUP50 and KPNB1, emphasizing its role in chromatin regulation [53]. MELTF was linked to NOTCH2NL and NBPF19, suggesting involvement in cellular differentiation pathways, while MECP2 indirectly interacted with chromatin remodelers such as SMARCA2 and YY1, known for their oncogenic roles in cancers, including leukemia [54].

Furthermore, PINA 3.0 network analysis (Figure 8B) further highlighted CRK as interacting with proteins linked to both poor and favorable AML prognoses. For instance, it positively interacted with SRC, HSPA6, and LMNA (poor prognosis) and negatively with KIT and ATXN1 (favorable prognosis). Moreover, GSPT1 interacted with ELAVL1 and EIF4B, proteins associated with favorable AML outcomes, while KPNA1 negatively interacted with PPP1CA, a marker of poor prognosis, suggesting its role in modulating negative prognostic pathways in leukemia. These findings are in line with the work of Li et al., who reported increased leukemia-associated gene expression in benzene-exposed workers [55].

Finally, miRNet disease pathway analysis identified nine key miRNAs involved in AML pathology: hsa-let-7b-5p, hsa-let-7a-5p, hsa-let-7d-5p, hsa-miR-124-3p, hsa-miR-128-3p, hsa-miR-15a-5p, hsa-miR-196b-5p, hsa-miR-19b-3p, and hsa-miR-210-3p. All these miRNAs concertedly regulate the post-transcriptional expression of the target genes MELTF, MECP2, KPNA1, CRK, and GSTP1 (Figure 9D). Pivotally, among these candidates, hsa-let-7d was previously found significantly overexpressed in the peripheral blood of benzene-exposed workers [56].

This study offers a comprehensive, multi-omics approach to understand the molecular impact of benzene exposure, leveraging an important contribution to the field of occupational toxicology. One notable strength lies in the integration of transcriptomic, epigenomic, functional pathway analyses, and interaction studies (i.e., protein-protein and miRNA-target interactions), showing a deeper insight into the complex molecular disruptions driven by benzene exposure. The identification of a conserved set of nine upregulated genes across multiple datasets underscores the robustness of the findings. Additionally, the inclusion of correlation assessments and ROC analyses adds a predictive dimension to the study, providing strong evidence that all nine genes, and particularly NFKB1, possess high discriminatory power in distinguishing exposed from non-exposed individuals. Such holistic evaluations not only strengthen the evidence base but also pave the way for the development of early detection biomarkers, which could significantly enhance occupational health surveillance.

However, this study also has limitations, primarily stemming from its reliance on publicly available datasets, which may introduce biases related to sample collection, handling, and cohort heterogeneity. Also, the discrepancy in gene expression data resulting from microarray and RNA-seq datasets exemplifies the technological variability that can affect the consistency of findings. Moreover, while the study successfully demonstrates differential gene expression and DNA methylation patterns, the cross-sectional nature of the datasets limits the ability to infer causality between benzene exposure and leukemogenesis. Another limitation is the lack of experimental validation in both preclinical and clinical settings, which is crucial to confirm the functional relevance of the identified biomarkers. Strategies such as immunohistochemistry and serum-based analyses are particularly effective for establishing correlations between benzene exposure and changes in gene expression, DNA methylation patterns, and miRNA levels, thereby enhancing the translational role of these findings [57,58].

In conclusion, by identifying a multi-omic molecular fingerprint, the findings of this study lay the groundwork for developing novel personalized prevention strategies for workers exposed to benzene. Once validated, these results could help tailor bio-monitoring approaches using specific biomarkers of gene expression, DNA methylation, and miRNA alterations to early identify at-risk workers. Furthermore, the integration of New Approach Methodologies (NAMs), which include not only omics applications but also machine learning models and high-throughput bioassays, could provide powerful tools for validating these findings and enhancing the human relevance of risk assessment frameworks, advancing toward next-generation, ethically sound toxicity testing paradigms [59,60]. This integrated approach could improve surveillance programs, enabling the prevention of long-term health risks associated with benzene exposure, even before the onset of clinical symptoms.

## 4. Materials and Methods

### 4.1. Selected Dataset Repositories, Gene Expression, and DNA Methylation Analyses

The Gene Expression Omnibus (GEO) DataSets public database was used to select gene expression datasets of individuals occupationally exposed to benzene and unexposed controls [61]. To evaluate the impact of benzene on exposed workers, an advanced search was performed using the following terms: “(benzene AND ‘Homo sapiens’ [porgn:txid9606])”. Only studies conducted in workers were selected, while preclinical data were excluded. A total of three datasets were shortlisted for computational analysis, as reported in Table S7. All datasets analyzed peripheral blood cell samples from workers exposed versus not exposed to benzene.

Additionally, datasets of samples from patients with acute myeloid leukemia (AML) and healthy controls were selected using the search string: “(‘AML’ OR ‘Acute Myeloid Leukemia’ AND ‘Homo sapiens’ [porgn:txid9606])”, with only clinical studies being included. Ten datasets were selected and analyzed (Table S8).

After downloading gene expression data matrices from GEO DataSets, differential analyses were performed to identify genetic and epigenetic factors deregulated by benzene. Normalized gene expression data were obtained using the GEO2R online bioinformatics tool (https://www.ncbi.nlm.nih.gov/geo/geo2r/, accessed on 19 December 2024) [62]. Samples were categorized into “non-exposed control group” and “exposed group”, or into “AML group” and “healthy group”, and differential analyses were conducted to compare the expression levels of each gene or methylation hotspot across datasets. The results were expressed as log_2_ fold change (log_2_FC). *p*-values were corrected for multiple testing using the Benjamini and Hochberg false discovery rate (FDR). FDR values ≤ 0.05 were considered statistically significant.

### 4.2. Functional Analyses of Candidate Genetic and Epigenetic Biomarkers

Gene enrichment analyses were performed using the g:Profiler online tool, (https://biit.cs.ut.ee/gprofiler/gost, accessed on 19 December 2024) specifically the g:GOSt function, which enables gene set enrichment analysis from Gene Ontology (GO), Kyoto Encyclopedia of Genes and Genomes (KEGG), and WikiPathways (WP) [63]. Additionally, the STRING v12.0 bioinformatics tool was used to analyze the interaction networks of differentially expressed genes [64].

The Human Reference Protein Interactome Mapping Project (HuRI) online tool was used to investigate human binary protein interactions. Specifically, the tissue-specific function was applied to identify genome-wide, tissue-preferentially expressed genes in whole blood [65]. The Protein Interaction Network Analysis (PINA 3.0) platform was also employed to examine cancer-specific interactomes. Results were filtered to include only direct interactions with correlation coefficients > 0.3 within the AML-specific network, which integrates data from TCGA-LAML [66].

To identify miRNAs regulating the expression of the identified genes, a miRNA regulatory network analysis was performed using the miRNet 2.0 online tool. Refinement filters (i.e., minimum network, Steiner-forest network, betweenness filter) were applied to enhance specificity [67].

### 4.3. Statistical Analyses and Plotting

Data processing and statistical analyses were conducted using GraphPad Prism version 9.0 for Windows (GraphPad Software, La Jolla, CA, USA). Results were presented as median ± standard deviation (SD), where applicable. Shapiro–Wilk normality tests were performed for each variable of interest, followed by appropriate statistical tests. Comparisons between two groups were made using the two-tailed unpaired or paired Student’s t-test or the Mann–Whitney U test. Comparisons between three or more groups were performed using one-way analysis of variance (ANOVA) or the Friedman test. The prognostic significance of deregulated genes was evaluated using receiver operating characteristic (ROC) curve analyses, with areas under the curve (AUC) calculated. Gene expression values were stratified into two classes (experiment vs. control) for each gene, and groups were analyzed using the ROC function in Prism.

Correlations between gene expressions were assessed using Spearman’s correlation coefficients, while the Mantel test was employed to measure linear relationships between distances. Corresponding correlograms were generated using ChiPlot (https://www.chiplot.online/#, accessed on 19 December 2024) [68]. Volcano plots, Venn diagrams, bubble plots, and heatmaps with associated clustering were generated using the SRplot online visualization platform (https://www.bioinformatics.com.cn/srplot, accessed on 19 December 2024) [69]. Statistical significance was defined as *p*-values < 0.05, with the following thresholds: * *p* < 0.05; ** *p* < 0.01; *** *p* < 0.001; and **** *p* < 0.0001.

## 5. Conclusions

This study identifies key genes as potential benzene biomarkers. NFKB1, crucial in inflammation and immune response, is consistently upregulated by benzene exposure. Similarly, MELTF, involved in iron metabolism and ferroptosis, links benzene exposure to hematotoxicity and AML through oxidative stress. Additionally, CRK and CXCR6 highlight early immune and oncogenic signaling disruptions, while ZNF331 underscores epigenetic changes. Also, KPNA1’s involvement in nuclear transport highlights cellular dysfunctions. Additionally, dose-dependent DNA methylation changes were identified in CXCR6 and MELTF, while specific miRNAs, including let-7d-5p, miR-126-3p, and miR-361-5p, were highlighted as crucial post-transcriptional regulators. Together, these genes and miRNAs form a robust biomarker panel, suggesting new opportunities for future preclinical and clinical validation.

Overall, our findings reinforce the value of applying a systems biology approach to occupational health, advocating for the integration of data from diverse datasets and the use of advanced network-based analyses to refine and confirm biomarkers of susceptibility and exposure. In fact, low-dose chronic benzene occupational exposure remains a significant concern, particularly in industrial settings; therefore, these insights offer a foundation for improved early detection and the development of targeted interventions to mitigate the long-term health effects of benzene harmfulness.

## Data Availability

The data generated in the present study may be requested from the corresponding author.

## Supplementary Materials

The supporting information can be downloaded at: https://www.mdpi.com/article/10.3390/ijms26031138/s1. References [70,71,72,73,74,75,76,77,78,79,80,81] are cited in the Supplementary Table S8.

## References

[1] Loomis D., Guyton K.Z., Grosse Y., El Ghissassi F., Bouvard V., Benbrahim-Tallaa L., Guha N., Vilahur N., Mattock H., Straif K. (2017). Carcinogenicity of benzene. Lancet Oncol..

[2] Wang J., Ma Y., Tang L., Li D., Xie J., Sun Y., Tian Y. (2024). Long-Term Exposure to Low Concentrations of Ambient Benzene and Mortality in a National English Cohort. Am. J. Respir. Crit. Care Med..

[3] Wang T., Cao Y., Xia Z., Christiani D.C., Au W.W. (2024). Review on novel toxicological effects and personalized health hazard in workers exposed to low doses of benzene. Arch. Toxicol..

[4] IARC Working Group on the Evaluation of Carcinogenic Risks to Humans (2018). Benzene—IARC Monographs on the Evaluation of Carcinogenic Risks to Humans, No. 120.

[5] European Parliament, Council of the European Union (2022). Directive (EU) 2022/431 of the European Parliament and of the Council of 9 March 2022 amending Directive 2004/37/EC on the protection of workers from the risks related to exposure to carcinogens or mutagens at work. Off. J. Eur. Union.

[6] Dewi R., Hamid Z.A., Rajab N., Shuib S., Razak S.A. (2020). Genetic, epigenetic, and lineage-directed mechanisms in benzene-induced malignancies and hematotoxicity targeting hematopoietic stem cells niche. Hum. Exp. Toxicol..

[7] Lu P.C.W., Shahbaz S., Winn L.M. (2020). Benzene and its effects on cell signaling pathways related to hematopoiesis and leukemia. J. Appl. Toxicol..

[8] He J., Peng C., Yang X., Li P., Bai J., Jia Q., Bo C. (2024). Identification of critical genes associated with oxidative stress pathways in benzene-induced hematotoxicity. Heliyon.

[9] Zhang Z., Shi W., Ru L., Lv W. (2024). Biomarkers of occupational benzene exposure: A Systematic Review to estimate the exposure levels and individual susceptibility at low doses. Toxicol. Ind. Health.

[10] McHale C.M., Smith M.T., Zhang L. (2016). Application of Transcriptomics in Exposed Human Populations: Benzene as an Example. Toxicogenomics in Predictive Carcinogenicity.

[11] Fenga C., Gangemi S., Costa C. (2016). Benzene exposure is associated with epigenetic changes (Review). Mol. Med. Rep..

[12] Spatari G., Allegra A., Carrieri M., Pioggia G., Gangemi S. (2021). Epigenetic Effects of Benzene in Hematologic Neoplasms: The Altered Gene Expression. Cancers.

[13] Zhang L., McHale C.M., Rothman N., Li G., Ji Z., Vermeulen R., Hubbard A.E., Ren X., Shen M., Rappaport S.M. (2010). Systems biology of human benzene exposure. Chem. Biol. Interact..

[14] Smith M.T., Vermeulen R., Li G., Zhang L., Lan Q., Hubbard A.E., Forrest M.S., McHale C., Zhao X., Gunn L. (2005). Use of ‘Omic’ technologies to study humans exposed to benzene. Chem. Biol. Interact..

[15] Sun R., Xu K., Ji S., Pu Y., Yu L., Yin L., Zhang J., Pu Y. (2021). Toxicity in hematopoietic stem cells from bone marrow and peripheral blood in mice after benzene exposure: Single-cell transcriptome sequencing analysis. Ecotoxicol. Environ. Saf..

[16] Fenga C., Gangemi S., Giambò F., Tsitsimpikou C., Golokhvast K., Tsatsakis A., Costa C. (2016). Low-dose occupational exposure to benzene and signal transduction pathways involved in the regulation of cellular response to oxidative stress. Life Sci..

[17] Boogaard P.J. (2022). Human biomonitoring of low-level benzene exposures. Crit. Rev. Toxicol..

[18] Forrest M.S., Lan Q., Hubbard A.E., Zhang L., Vermeulen R., Zhao X., Li G., Wu Y.-Y., Shen M., Yin S. (2005). Discovery of Novel Biomarkers by Microarray Analysis of Peripheral Blood Mononuclear Cell Gene Expression in Benzene-Exposed Workers. Environ. Health Perspect..

[19] Park T. (2021). Crk and CrkL as Therapeutic Targets for Cancer Treatment. Cells.

[20] Shen Q., Bhatt V.S., Krieger I., Sacchettini J.C., Cho J.-H. (2018). Structure-guided design of a potent peptide inhibitor targeting the interaction between CRK and ABL kinase. Medchemcomm.

[21] Nakasone E.S., Zemla T.J., Yu M., Lin S.Y., Ou F.-S., Carter K., Innocenti F., Saltz L., Grady W.M., Cohen S.A. (2024). Evaluating the utility of ZNF331 promoter methylation as a prognostic and predictive marker in stage III colon cancer: Results from CALGB 89803 (Alliance). Epigenetics.

[22] Nie C., Han X., Wei R., Leonteva A., Hong J., Du X., Wang J., Zhu L., Zhao Y., Xue Y. (2021). Association of ZNF331 and WIF1 methylation in peripheral blood leukocytes with the risk and prognosis of gastric cancer. BMC Cancer.

[23] Bao N., Fu B., Zhong X., Jia S., Ren Z., Wang H., Wang W., Shi H., Li J., Ge F. (2023). Role of the CXCR6/CXCL16 axis in autoimmune diseases. Int. Immunopharmacol..

[24] Korbecki J., Kupnicka P., Barczak K., Bosiacki M., Ziętek P., Chlubek D., Baranowska-Bosiacka I. (2023). The Role of CXCR1, CXCR2, CXCR3, CXCR5, and CXCR6 Ligands in Molecular Cancer Processes and Clinical Aspects of Acute Myeloid Leukemia (AML). Cancers.

[25] Schiffman C., McHale C.M., Hubbard A.E., Zhang L., Thomas R., Vermeulen R., Li G., Shen M., Rappaport S.M., Yin S. (2018). Identification of gene expression predictors of occupational benzene exposure. PLoS ONE.

[26] Thomas R., McHale C.M., Lan Q., Hubbard A.E., Zhang L., Vermeulen R., Li G., Rappaport S.M., Yin S., Rothman N. (2013). Global gene expression response of a population exposed to benzene: A pilot study exploring the use of RNA-sequencing technology. Environ. Mol. Mutagen..

[27] van der Kloet F.M., Buurmans J., Jonker M.J., Smilde A.K., Westerhuis J.A. (2020). Increased comparability between RNA-Seq and microarray data by utilization of gene sets. PLoS Comput. Biol..

[28] Pyatt D.W., Stillman W.S., Irons R.D. (1998). Hydroquinone, a Reactive Metabolite of Benzene, Inhibits NF-κB in Primary Human CD4+T Lymphocytes. Toxicol. Appl. Pharmacol..

[29] Zolghadr F., Sadeghizadeh M., Amirizadeh N., Hosseinkhani S., Nazem S. (2012). How benzene and its metabolites affect human marrow derived mesenchymal stem cells. Toxicol. Lett..

[30] Guo Q., Jin Y., Chen X., Ye X., Shen X., Lin M., Zeng C., Zhou T., Zhang J. (2024). NF-κB in biology and targeted therapy: New insights and translational implications. Signal Transduct. Target. Ther..

[31] Mao Y., Wang M., Xiong Y., Wen X., Zhang M., Ma L., Zhang Y. (2023). MELTF Might Regulate Ferroptosis, Pyroptosis, and Autophagy in Platelet-Rich Plasma-Mediated Endometrial Epithelium Regeneration. Reprod. Sci..

[32] Sun R., Liu M., Xu K., Pu Y., Huang J., Liu J., Zhang J., Yin L., Pu Y. (2022). Ferroptosis is involved in the benzene-induced hematotoxicity in mice via iron metabolism, oxidative stress and NRF2 signaling pathway. Chem. Biol. Interact..

[33] Rothman N., Vermeulen R., Zhang L., Hu W., Yin S., Rappaport S.M., Smith M.T., Jones D.P., Rahman M., Lan Q. (2021). Metabolome-wide association study of occupational exposure to benzene. Carcinogenesis.

[34] Liang B., Chen Y., Yuan W., Qin F., Zhang Q., Deng N., Liu X., Ma X., Zhang X., Zhang B. (2018). Down-regulation of miRNA-451a and miRNA-486-5p involved in benzene-induced inhibition on erythroid cell differentiation in vitro and in vivo. Arch. Toxicol..

[35] Liu Y., Chen X., Bian Q., Shi Y., Liu Q., Ding L., Zhang H., Zhu B. (2016). Analysis of plasma microRNA expression profiles in a Chinese population occupationally exposed to benzene and in a population with chronic benzene poisoning. J. Thorac. Dis..

[36] Rahmanto Y.S., Bal S., Loh K.H., Yu Y., Richardson D.R. (2012). Melanotransferrin: Search for a function. Biochim. Biophys. Acta-Gen. Subj..

[37] Sawaki K., Kanda M., Umeda S., Miwa T., Tanaka C., Kobayashi D., Hayashi M., Yamada S., Nakayama G., Omae K. (2019). Level of Melanotransferrin in Tissue and Sera Serves as a Prognostic Marker of Gastric Cancer. Anticancer Res..

[38] Shin J., Kim H.-J., Kim G., Song M., Woo S.J., Lee S.-T., Kim H., Lee C. (2014). Discovery of Melanotransferrin as a Serological Marker of Colorectal Cancer by Secretome Analysis and Quantitative Proteomics. J. Proteome Res..

[39] Huang Y., Clarke F., Karimi M., Roy N.H., Williamson E.K., Okumura M., Mochizuki K., Chen E.J.H., Park T.-J., Debes G.F. (2015). CRK proteins selectively regulate T cell migration into inflamed tissues. J. Clin. Investig..

[40] Zarth A.T., Murphy S.E., Hecht S.S. (2015). Benzene oxide is a substrate for glutathione S-transferases. Chem. Biol. Interact..

[41] Nourozi M.A., Neghab M., Bazzaz J.T., Nejat S., Mansoori Y., Shahtaheri S.J. (2018). Association between polymorphism of GSTP1, GSTT1, GSTM1 and CYP2E1 genes and susceptibility to benzene-induced hematotoxicity. Arch. Toxicol..

[42] Zhang D., Lin P., Lin J. (2024). Molecular glues targeting GSPT1 in cancers: A potent therapy. Bioorg. Chem..

[43] Pierce D.W., Yao T.-W.S., Pace E., Wang H., Flandin-Blety P., Benitez A., Guarinos C., Hoffmann M., Carrancio S., Fan J. (2021). Synergistic Combination Activity of the Novel GSPT1 Degrader CC-90009 in Acute Myeloid Leukemia Models. Blood.

[44] Chang Y., Keramatnia F., Ghate P.S., Nishiguchi G., Gao Q., Iacobucci I., Yang L., Chepyala D., Mishra A., High A.A. (2023). The orally bioavailable GSPT1/2 degrader SJ6986 exhibits in vivo efficacy in acute lymphoblastic leukemia. Blood.

[45] Keramatnia F., Chang Y., Nishiguchi G., Min J., Mullighan C., Fischer M., Rankovic Z., Keramatnia F. (2021). Abstract LBA002: Targeting GSPT1 by a novel cereblon E3 ligase modulator for the treatment of Acute Lymphoblastic Leukemia. Mol. Cancer Ther..

[46] Newell S., van der Watt P.J., Leaner V.D. (2024). Therapeutic targeting of nuclear export and import receptors in cancer and their potential in combination chemotherapy. IUBMB Life.

[47] Kramer M.H., Zhang Q., Sprung R., Day R.B., Erdmann-Gilmore P., Li Y., Xu Z., Helton N.M., George D.R., Mi Y. (2022). Proteomic and phosphoproteomic landscapes of acute myeloid leukemia. Blood.

[48] Derecki N.C., Cronk J.C., Lu Z., Xu E., Abbott S.B.G., Guyenet P.G., Kipnis J. (2012). Wild-type microglia arrest pathology in a mouse model of Rett syndrome. Nature.

[49] Nejati-Koshki K., Roberts C.-T., Babaei G., Rastegar M. (2023). The Epigenetic Reader Methyl-CpG-Binding Protein 2 (MeCP2) Is an Emerging Oncogene in Cancer Biology. Cancers.

[50] Frietsch J.J., Kastner C., Grunewald T.G.P., Schweigel H., Nollau P., Ziermann J., Clement J.H., La Rosée P., Hochhaus A., Butt E. (2014). LASP1 is a novel BCR-ABL substrate and a phosphorylation-dependent binding partner of CRKL in chronic myeloid leukemia. Oncotarget.

[51] Cao M., Carrasco R.D., Dubuc A.M., Cin P.D., Fletcher J.A., Xiao S. (2019). ZMYM2-FGFR1 fusion as secondary change in acute myeloid leukemia. Leuk. Lymphoma.

[52] Sellar R.S., Sperling A.S., Słabicki M., Gasser J.A., McConkey M.E., Donovan K.A., Mageed N., Adams D.N., Zou C., Miller P.G. (2022). Degradation of GSPT1 causes TP53-independent cell death in leukemia while sparing normal hematopoietic stem cells. J. Clin. Investig..

[53] Choo H.-J., Cutler A., Rother F., Bader M., Pavlath G.K. (2016). Karyopherin Alpha 1 Regulates Satellite Cell Proliferation and Survival by Modulating Nuclear Import. Stem Cells.

[54] Noguera N.I., Travaglini S., Scalea S., Catalanotto C., Reale A., Zampieri M., Zaza A., Ricciardi M.R., Angelini D.F., Tafuri A. (2023). YY1 Knockdown Relieves the Differentiation Block and Restores Apoptosis in AML Cells. Cancers.

[55] Li K., Jing Y., Yang C., Liu S., Zhao Y., He X., Li F., Han J., Li G. (2014). Increased leukemia-associated gene expression in benzene-exposed workers. Sci. Rep..

[56] Bai W., Chen Y., Yang J., Niu P., Tian L., Gao A. (2014). Aberrant miRNA profiles associated with chronic benzene poisoning. Exp. Mol. Pathol..

[57] Li P., Wu Y., Zhang Z., Lin D., Wang D., Huang X., Zhang Y. (2019). Proteomics analysis identified serum biomarkers for occupational benzene exposure and chronic benzene poisoning. Medicine.

[58] Liang B., Zhong Y., Chen K., Zeng L., Li G., Zheng J., Jiang L., Xie Z., Que B., Lai G. (2018). Serum plasminogen as a potential biomarker for the effects of low-dose benzene exposure. Toxicology.

[59] Schmeisser S., Miccoli A., von Bergen M., Berggren E., Braeuning A., Busch W., Desaintes C., Gourmelon A., Grafström R., Harrill J. (2023). New approach methodologies in human regulatory toxicology—Not if, but how and when!. Environ. Int..

[60] Faulhammer F., van Ravenzwaay B., Schnatter A.R., Rooseboom M., Kamp H., Flick B., Giri V., Sperber S., Higgins L.G., Penman M.G. (2024). The short-term toxicity and metabolome of Benzene. Toxicol. Lett..

[61] GEO DataSets NIH. https://www.ncbi.nlm.nih.gov/gds.

[62] Clough E., Barrett T., Wilhite S.E., Ledoux P., Evangelista C., Kim I.F., Tomashevsky M., Marshall K.A., Phillippy K.H., Sherman P.M. (2024). NCBI GEO: Archive for gene expression and epigenomics data sets: 23-year update. Nucleic Acids Res..

[63] Kolberg L., Raudvere U., Kuzmin I., Adler P., Vilo J., Peterson H. (2023). g: Profiler—Interoperable web service for functional enrichment analysis and gene identifier mapping (2023 update). Nucleic Acids Res..

[64] Szklarczyk D., Kirsch R., Koutrouli M., Nastou K., Mehryary F., Hachilif R., Gable A.L., Fang T., Doncheva N.T., Pyysalo S. (2023). The STRING database in 2023: Protein–protein association networks and functional enrichment analyses for any sequenced genome of interest. Nucleic Acids Res..

[65] Luck K., Kim D.-K., Lambourne L., Spirohn K., Begg B.E., Bian W., Brignall R., Cafarelli T., Campos-Laborie F.J., Charloteaux B. (2020). A reference map of the human binary protein interactome. Nature.

[66] Du Y., Cai M., Xing X., Ji J., Yang E., Wu J. (2021). PINA 3.0: Mining cancer interactome. Nucleic Acids Res..

[67] Chang L., Xia J. (2023). MicroRNA Regulatory Network Analysis Using miRNet 2.0. Transcription Factor Regulatory Networks.

[68] ChiPlot—A Web-Based Tool for Free Bioinformatics Plots. https://www.chiplot.online/.

[69] Tang D., Chen M., Huang X., Zhang G., Zeng L., Zhang G., Wu S., Wang Y. (2023). SRplot: A free online platform for data visualization and graphing. PLoS ONE.

[70] McHale C.M., Zhang L., Lan Q., Vermeulen R., Li G., Hubbard A.E., Porter K.E., Thomas R., Portier C.J., Shen M. (2011). Global Gene Expression Profiling of a Population Exposed to a Range of Benzene Levels. Environ. Health Perspect..

[71] McHale C.M., Zhang L., Lan Q., Li G., Hubbard A.E., Forrest M.S., Vermeulen R., Chen J., Shen M., Rappaport S.M. (2009). Changes in the Peripheral Blood Transcriptome Associated with Occupational Benzene Exposure Identified by Cross-Comparison on Two Microarray Platforms. Genomics.

[72] Bai W., Yang J., Yang G., Niu P., Tian L., Gao A. (2014). Long Non-Coding RNA NR_045623 and NR_028291 Involved in Benzene Hematotoxicity in Occupationally Benzene-Exposed Workers. Exp. Mol. Pathol..

[73] Chen Y., Hoffmeister L.M., Zaun Y., Arnold L., Schmid K.W., Giebel B., Klein-Hitpass L., Hanenberg H., Squire A., Reinhardt H.C. (2020). Acute Myeloid Leukemia–Induced Remodeling of the Human Bone Marrow Niche Predicts Clinical Outcome. Blood Adv..

[74] Huang H.-H., Chen F.-Y., Chou W.-C., Hou H.-A., Ko B.-S., Lin C.-T., Tang J.-L., Li C.-C., Yao M., Tsay W. (2019). Long Non-Coding RNA HOXB-AS3 Promotes Myeloid Cell Proliferation and Its Higher Expression Is an Adverse Prognostic Marker in Patients with Acute Myeloid Leukemia and Myelodysplastic Syndrome. BMC Cancer.

[75] Boyd A.L., Reid J.C., Salci K.R., Aslostovar L., Benoit Y.D., Shapovalova Z., Nakanishi M., Porras D.P., Almakadi M., Campbell C.J.V. (2017). Acute Myeloid Leukaemia Disrupts Endogenous Myelo-Erythropoiesis by Compromising the Adipocyte Bone Marrow Niche. Nat. Cell Biol..

[76] Li K., Wang F., Cao W.-B., Lv X.-X., Hua F., Cui B., Yu J.-J., Zhang X.-W., Shang S., Liu S.-S. (2017). TRIB3 Promotes APL Progression through Stabilization of the Oncoprotein PML-RARα and Inhibition of P53-Mediated Senescence. Cancer Cell.

[77] von der Heide E.K., Neumann M., Vosberg S., James A.R., Schroeder M.P., Ortiz-Tanchez J., Isaakidis K., Schlee C., Luther M., Jöhrens K. (2017). Molecular Alterations in Bone Marrow Mesenchymal Stromal Cells Derived from Acute Myeloid Leukemia Patients. Leukemia.

[78] Tan S.-F., Liu X., Fox T.E., Barth B.M., Sharma A., Turner S.D., Awwad A., Dewey A., Doi K., Spitzer B. (2016). Acid Ceramidase Is Upregulated in AML and Represents a Novel Therapeutic Target. Oncotarget.

[79] Le Dieu R., Taussig D.C., Ramsay A.G., Mitter R., Miraki-Moud F., Fatah R., Lee A.M., Lister T.A., Gribben J.G. (2009). Peripheral Blood T Cells in Acute Myeloid Leukemia (AML) Patients at Diagnosis Have Abnormal Phenotype and Genotype and Form Defective Immune Synapses with AML Blasts. Blood.

[80] Goswami M., Prince G., Biancotto A., Moir S., Kardava L., Santich B.H., Cheung F., Kotliarov Y., Chen J., Shi R. (2017). Impaired B Cell Immunity in Acute Myeloid Leukemia Patients after Chemotherapy. J. Transl. Med..

[81] Knaus H.A., Berglund S., Hackl H., Blackford A.L., Zeidner J.F., Montiel-Esparza R., Mukhopadhyay R., Vanura K., Blazar B.R., Karp J.E. (2018). Signatures of CD8+ T Cell Dysfunction in AML Patients and Their Reversibility with Response to Chemotherapy. JCI Insight.

