# Supplementary material for: Integrated Computational Analysis Reveals Early Genetic and Epigenetic AML Susceptibility Biomarkers in Benzene-Exposed Workers
Source: Int J Mol Sci. 2025 Jan 28;26(3):1138. doi: 10.3390/ijms26031138 (PMC11818736; doi:10.3390/ijms26031138)
Supplement: Supplementary file 1 [file ijms-26-01138-s001.zip › ijms-3413613-supplementary.pdf]

*Supplementary Material*

# Integrated Computational Analysis Reveals Early Genetic and Epigenetic AML Susceptibility Biomarkers in Benzene-Exposed Workers

By Silvia Vivarelli [1,*] , Cigdem Sevim [2], Federica Giambò [1] and Concettina Fenga [1]
(Correspondence: silvia.vivarelli@unime.it)

## SUPPLEMENTARY FIGURES

**Figure S1**

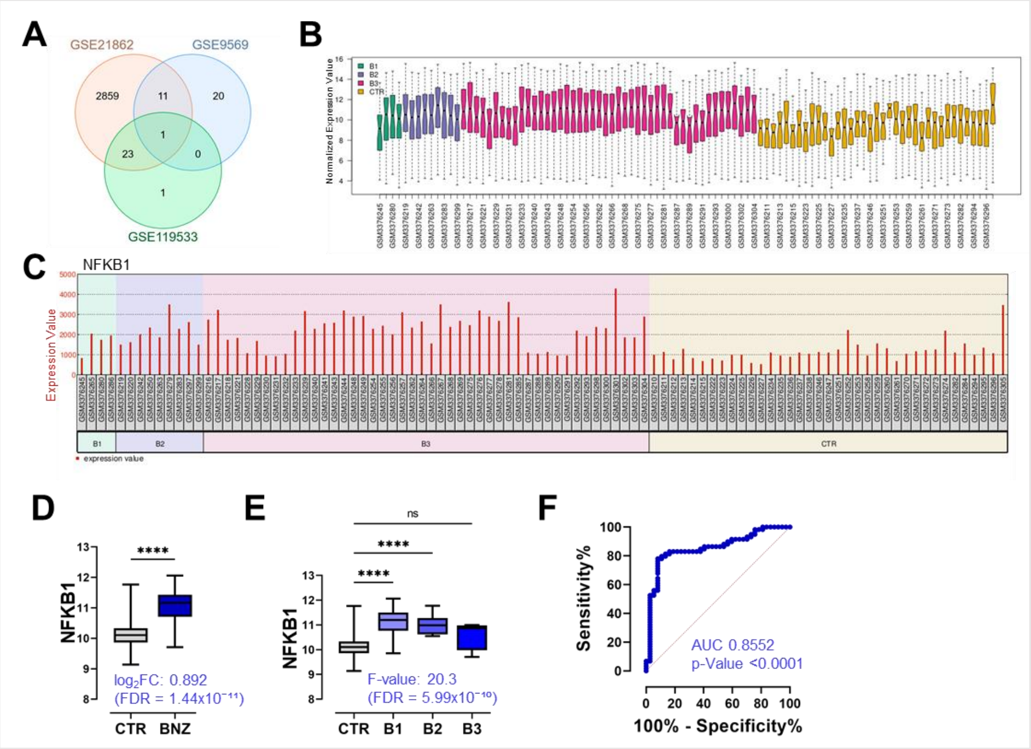


**Figure S1.** Validation of NFKB1 expression in an independent dataset (GSE119533). **(A)** Venn diagram of significantly deregulated genes in GSE119533, GSE21862, GSE9569. **(B)** Normalized expression values of genes in GSE119533. **(C)** Expression value distribution for NFKB1 in GSE119533. B1 (benzene <0.1 ppm), B2 (benzene 5–10 ppm), and B3 (benzene >10 ppm); CTR (non-exposed controls). **(D)** and **(E)** Box plots showing the median expression levels ± SD of CTR (N=37) and BNZ (N=59) in C and of CTR (N=37), B1 (N=46), B2 (N=9), B3 (N=4). **(F)** ROC analysis with AUC. **** $p < 0.0001$; ns = not significant.

**Figure S2**

**A**

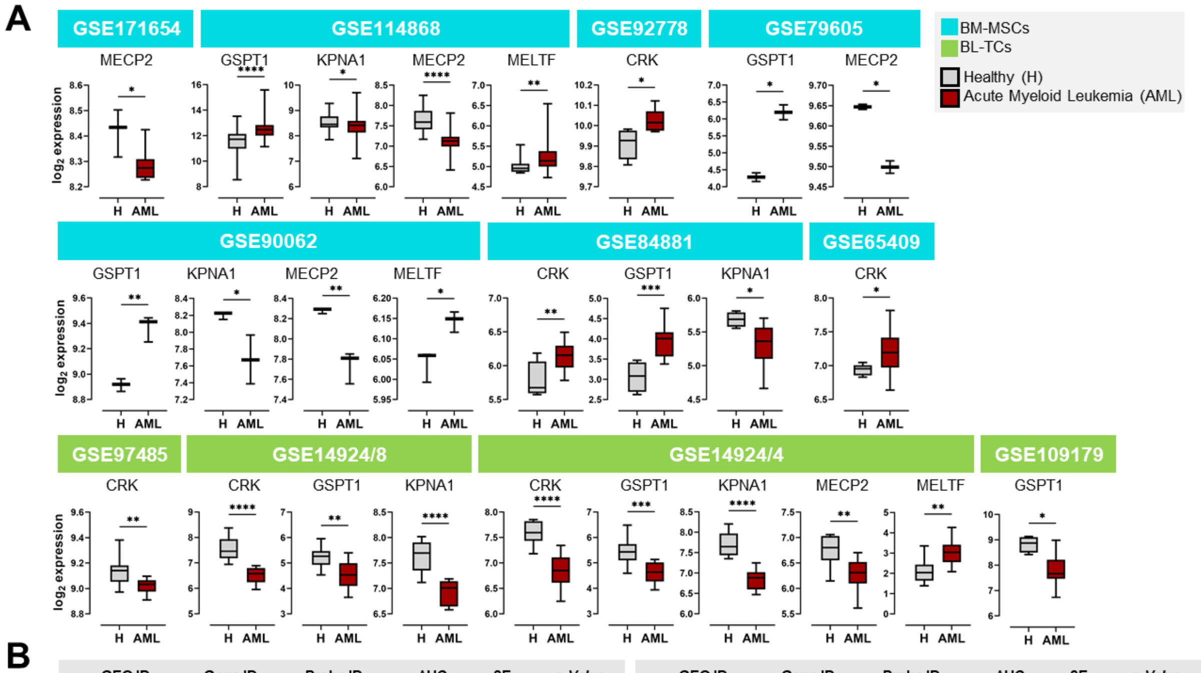


**B**

| GEO ID | Gene ID | Probe ID | AUC | SE | *p-Value* |
|---|---|---|---|---|---|
| GSE171654 | *MECP2* | TC0X00011185.hg.1 | 0.958 | 0.061 | *0.0247* |
| GSE114868 | *GSPT1* | TC16000875.hg.1 | 0.779 | 0.062 | *<0.0001* |
| | *KPNA1* | TC03001704.hg.1 | 0.628 | 0.063 | *0.0605* |
| | *MECP2* | TC0X001525.hg.1 | 0.941 | 0.025 | *<0.0001* |
| | *MELTF* | TC03002173.hg.1 | 0.745 | 0.054 | *0.0003* |
| GSE92778 | *CRK* | s_8011018 | 0.917 | 0.081 | *0.0163* |
| GSE90062 | *GSPT1* | 11718222_s_at | na | na | *na* |
| | *KPNA1* | 11734667_a_at | na | na | *na* |
| | *MECP2* | 11722683_a_at | na | na | *na* |
| | *MELTF* | 11736415_at | na | na | *na* |
| GSE84881 | *CRK* | 202224_at | 0.855 | 0.131 | *0.0285* |
| | *GSPT1* | 240452_at | 0.987 | 0.020 | *0.0027* |
| | *KPNA1* | 213741_s_at | 0.895 | 0.075 | *0.0150* |
| GSE79605 | *GSPT1* | A_23_P206301 | na | na | *na* |
| | *MECP2* | A_24_P237486 | na | na | *na* |
| GSE65409 | *CRK* | ILMN_1666494 | 0.808 | 0.068 | *0.0081* |

| GEO ID | Gene ID | Probe ID | AUC | SE | *p-Value* |
|---|---|---|---|---|---|
| GSE97485 | *CRK* | s_8011018 | 0.820 | 0.103 | *0.0156* |
| GSE14924/8 | *CRK* | 202226_s_at | 1.000 | 0.000 | *0.0002* |
| | *GSPT1* | 217595_at | 0.850 | 0.088 | *0.0082* |
| | *KPNA1* | 202057_at | 0.970 | 0.035 | *0.0004* |
| GSE14924/4 | *CRK* | 202226_s_at | 0.980 | 0.026 | *0.0003* |
| | *GSPT1* | 217595_at | 0.890 | 0.077 | *0.0032* |
| | *KPNA1* | 202057_at | 1.000 | 0.000 | *0.0002* |
| | *MECP2* | 202617_s_at | 0.840 | 0.093 | *0.0102* |
| | *MELTF* | 220043_s_at | 0.840 | 0.095 | *0.0102* |
| GSE109179 | *GSPT1* | 11718222_s_at | 0.944 | 0.065 | *0.0136* |

**Figure S2.** Expression value distribution for CRK, GSPT1, KPNA1, MECP2, and MELTF in AML GEO datasets. **(A)** Box plots showing the median expression levels ± SD in Healthy (H) and Acute Myeloid Leukemia (AML) samples. **(B)** Table summarizing the ROC analysis values (Area Under the Curve, AUC; Standard Error, SE; and p-Value) for each gene across all datasets. The AUC values indicate the discriminatory power of the genes, with higher AUCs reflecting stronger predictive capability in distinguishing AML from healthy samples. * $p < 0.05$; ** $p < 0.01$; *** $p < 0.001$; and **** $p < 0.0001$.

## SUPPLEMENTARY TABLES

**Table S1. Significantly deregulated genes in benzene-exposed versus non-exposed workers.**

| | | | GSE21862 | | | GSE9569 | | |
|---|---|---|---|---|---|---|---|---|
| **Gene Name** | **Gene Title** | **Gene ID** | **Probe ID** | **logFC** | **FDR** | **Probe ID** | **logFC** | **FDR** |
| CD69 | CD69 molecule | 969 | ILMN_1651316 | 0.328 | $4.06X10^{-2}$ | 237009_at | -1.946 | $6.69X10^{-3}$ |
| CRK | CRK proto-oncogene, adaptor protein | 1398 | ILMN_1803302 | 0.128 | $7.25X10^{-3}$ | 202224_at | 0.499 | $3.70X10^{-2}$ |
| CXCR6 | C-X-C motif chemokine receptor 6 | 10663 | ILMN_1674640 | 0.247 | $2.85X10^{-3}$ | 206974_at | 0.566 | $2.88X10^{-2}$ |
| GSPT1 | G1 to S phase transition 1 | 2935 | ILMN_1750130 | 0.193 | $2.39X10^{-3}$ | 225276_at | 0.895 | $4.57X10^{-2}$ |
| JUN | Jun proto-oncogene, AP-1 transcription factor subunit | 3725 | ILMN_1669768 | 0.325 | $3.79X10^{-3}$ | 201466_s_at | -1.412 | $1.31X10^{-2}$ |
| KLF6 | Kruppel like factor 6 | 1316 | ILMN_1702995 | 0.252 | $6.24X10^{-3}$ | 208961_s_at | -1.048 | $3.43X10^{-2}$ |
| KPNA1 | karyopherin subunit alpha 1 | 3836 | ILMN_1751859 | 0.149 | $1.45X10^{-4}$ | 202055_at | 0.591 | $3.01X10^{-2}$ |
| MECP2 | methyl-CpG binding protein 2 | 4204 | ILMN_1682091 | 0.237 | $2.92X10^{-6}$ | 202617_s_at | 0.631 | $3.43X10^{-2}$ |
| MELTF | melanotransferrin | 4241 | ILMN_1746649 | 0.171 | $3.83X10^{-4}$ | 223723_at | 0.626 | $4.57X10^{-2}$ |
| NFKB1 | nuclear factor kappa B subunit 1 | 4790 | ILMN_1714965 | 0.577 | $8.54X10^{-9}$ | 209239_at | 0.812 | $4.60X10^{-2}$ |
| TBC1D7 | TBC1 domain family member 7 | 51256 | ILMN_1661622 | 0.368 | $5.20X10^{-5}$ | 223461_at | 0.628 | $1.60X10^{-2}$ |
| ZNF331 | zinc finger protein 331 | 55422 | ILMN_1711199 | 0.357 | $2.60X10^{-3}$ | 219228_at | 0.848 | $1.31X10^{-2}$ |

**Table S2. Mantel test results on genes in benzene exposed workers expression datasets.**

| | **Gene** | **Mantel r** | ***P-value*** |
|---|---|---|---|
| **CTR GSE21862** | CRK | 0.600 | *0.001* |
| | CXCR6 | 0.317 | *0.006* |
| | GSPT1 | 0.437 | *0.001* |
| | KPNA1 | 0.336 | *0.009* |
| | MECP2 | 0.564 | *0.001* |
| | MELTF | 0.621 | *0.001* |
| | NFKB1 | 0.811 | *0.001* |
| | TBC1D7 | 0.586 | *0.001* |
| | ZNF331 | 0.464 | *0.001* |
| **BZN GSE21862** | CRK | 0.080 | *0.035* |
| | CXCR6 | 0.309 | *0.001* |
| | GSPT1 | 0.585 | *0.001* |
| | KPNA1 | 0.280 | *0.001* |
| | MECP2 | 0.332 | *0.001* |
| | MELTF | 0.367 | *0.001* |
| | NFKB1 | 0.767 | *0.001* |
| | TBC1D7 | 0.576 | *0.001* |
| | ZNF331 | 0.738 | *0.001* |
| **CTR GSE9569** | CRK | 0.272 | *0.167* |
| | CXCR6 | 0.287 | *0.107* |
| | GSPT1 | 0.507 | *0.004* |
| | KPNA1 | 0.162 | *0.186* |
| | MECP2 | 0.443 | *0.025* |
| | MELTF | 0.170 | *0.275* |
| | NFKB1 | 0.659 | *0.002* |
| | TBC1D7 | 0.206 | *0.223* |
| | ZNF331 | 0.057 | *0.432* |

| | | | |
|---|---|---|---|
| BZN GSE9569 | CRK | -0.094 | *0.665* |
| | CXCR6 | 0.555 | *0.018* |
| | GSPT1 | 0.648 | *0.001* |
| | KPNA1 | 0.322 | *0.058* |
| | MECP2 | 0.020 | *0.435* |
| | MELTF | 0.144 | *0.275* |
| | NFKB1 | 0.531 | *0.024* |
| | TBC1D7 | 0.526 | *0.014* |
| | ZNF331 | 0.497 | *0.008* |

*In red: significant p-Values (p>0.05)

**Table S3. Enrichment network analysis of STRING data.**

| Category | Term ID | Term Description | strength | signal | FDR | Matching Proteins |
|---|---|---|---|---|---|---|
| GO Process | GO:0006607 | NLS-bearing protein import into nucleus | 2.19 | 0.77 | 0.0208 | KPNA3,KPNA4,**KPNA1** |
| GO Function | GO:0061608 | Nuclear import signal receptor activity | 2.21 | 1.02 | 0.0057 | KPNA3,KPNA4,**KPNA1** |
| GO Function | GO:0008139 | Nuclear localization sequence binding | 2.08 | 0.98 | 0.0067 | KPNA3,KPNA4,**KPNA1** |
| GO Component | GO:0042564 | NLS-dependent protein nuclear import complex | 2.54 | 1.59 | 0.0003 | KPNA3,KPNA4,**KPNA1** |
| STRING clusters | CL:2707 | Nucleocytoplasmic carrier activity, and Exportin-2, central domain | 2.09 | 0.88 | 0.0112 | KPNA3,KPNA4,**KPNA1** |
| STRING clusters | CL:2721 | NLS-dependent protein nuclear import complex | 2.62 | 0.78 | 0.0211 | KPNA3,KPNA4 |
| KEGG | hsa05132 | Salmonella infection | 1.39 | 1.18 | 0.0005 | **NFKB1**,KPNA3,KPNA4,**KPNA1**,MAP2K4 |
| KEGG | hsa04062 | Chemokine signaling pathway | 1.35 | 0.87 | 0.0049 | **NFKB1**,CXCL3,**CRK**,**CXCR6** |
| KEGG | hsa05120 | Epithelial cell signaling in Helicobacter pylori infection | 1.68 | 0.97 | 0.0049 | **NFKB1**,CXCL3,MAP2K4 |
| KEGG | hsa04668 | TNF signaling pathway | 1.45 | 0.75 | 0.0143 | **NFKB1**,CXCL3,MAP2K4 |
| KEGG | hsa05135 | Yersinia infection | 1.4 | 0.72 | 0.0158 | **NFKB1**,**CRK**,MAP2K4 |
| KEGG | hsa05167 | Kaposi sarcoma-associated herpesvirus infection | 1.22 | 0.54 | 0.0426 | **NFKB1**,CXCL3,MAP2K4 |
| Reactome | HSA-168276 | NS1 Mediated Effects on Host Pathways | 1.88 | 0.74 | 0.0224 | KPNA3,KPNA4,**KPNA1** |
| Reactome | HSA-1169408 | ISG15 antiviral mechanism | 1.62 | 0.59 | 0.0449 | KPNA3,KPNA4,**KPNA1** |
| Reactome | HSA-1280215 | Cytokine Signaling in Immune system | 0.95 | 0.46 | 0.0449 | **NFKB1**,KPNA3,**CRK**,KPNA4,**KPNA1**,MAP2K4 |
| WikiPathways | WP3929 | Chemokine signaling pathway | 1.4 | 0.74 | 0.0143 | **NFKB1**,CXCL3,**CRK**,**CXCR6** |
| COMPARTMENTS | GOCC:0042564 | NLS-dependent protein nuclear import complex | 2.54 | 1.57 | 0.0004 | KPNA3,KPNA4,**KPNA1** |
| Pfam | PF01749 | Importin beta binding domain | 2.65 | 1.7 | 0.0002 | KPNA3,KPNA4,**KPNA1** |
| Pfam | PF16186 | Atypical Arm repeat | 2.65 | 1.7 | 0.0002 | KPNA3,KPNA4,**KPNA1** |
| InterPro | IPR002652 | Importin-alpha, importin-beta-binding domain | 2.54 | 1.47 | 0.0007 | KPNA3,KPNA4,**KPNA1** |

| | | | | | | |
|---|---|---|---|---|---|---|
| InterPro | IPR024931 | Importin subunit alpha | 2.65 | 1.47 | 0.0007 | KPNA3,KPNA4,**KPNA1** |
| InterPro | IPR032413 | Atypical Arm repeat | 2.65 | 1.47 | 0.0007 | KPNA3,KPNA4,**KPNA1** |
| InterPro | IPR036975 | Importin-alpha, importin-beta-binding domain superfamily | 2.65 | 1.47 | 0.0007 | KPNA3,KPNA4,**KPNA1** |
| InterPro | IPR000225 | Armadillo | 1.86 | 0.8 | 0.0161 | KPNA3,KPNA4,**KPNA1** |
| SMART | SM00185 | Armadillo/beta-catenin-like repeats | 2.03 | 1.12 | 0.0030 | KPNA3,KPNA4,**KPNA1** |

*In red: query proteins

**Table S4. List of protein-protein interactions generated using the Human Reference Interactome (HuRI) in blood tissue.**

| Interactor A Gene Name | Interactor B Gene Name | Interactor A Ensembl ID | Interactor B Ensembl ID | Interactor A Uniprot ID | Interactor B Uniprot ID |
|---|---|---|---|---|---|
| ABL1 | CBL | ENSG00000097007 | ENSG00000110395 | P00519-2 | P22681 |
| ABL1 | GRB2 | ENSG00000097007 | ENSG00000177885 | P00519-2 | P62993-1 |
| ABL1 | MAP4K1 | ENSG00000097007 | ENSG00000104814 | P00519-2 | Q92918-1 |
| ABL1 | PIK3R1 | ENSG00000097007 | ENSG00000145675 | P00519-2 | P27986-2 |
| ABL1 | RAPGEF1 | ENSG00000097007 | ENSG00000107263 | P00519-2 | Q13905-4 |
| ABL1 | SRC | ENSG00000097007 | ENSG00000197122 | P00519-2 | P12931-1 |
| ABL2 | GRB2 | ENSG00000143322 | ENSG00000177885 | P42684-3 | P62993-1 |
| ASAP1 | GRB2 | ENSG00000153317 | ENSG00000177885 | Q9ULH1 | P62993-1 |
| ASAP1 | SRC | ENSG00000153317 | ENSG00000197122 | Q9ULH1 | P12931-1 |
| ATXN1 | ATXN1 | ENSG00000124788 | ENSG00000124788 | P54253-1 | P54253-1 |
| ATXN1 | TCAP | ENSG00000124788 | ENSG00000173991 | P54253-1 | O15273 |
| BEX5 | BEX5 | ENSG00000184515 | ENSG00000184515 | Q5H9J7 | Q5H9J7 |
| CBL | CBL | ENSG00000110395 | ENSG00000110395 | P22681 | P22681 |
| CBL | GRB2 | ENSG00000110395 | ENSG00000177885 | P22681 | P62993-1 |
| CBL | PIK3R1 | ENSG00000110395 | ENSG00000145675 | P22681 | P27986-2 |
| CBL | PIK3R3 | ENSG00000110395 | ENSG00000117461 | P22681 | Q92569-1 |
| CBL | SRC | ENSG00000110395 | ENSG00000197122 | P22681 | P12931-1 |
| CBLB | GRB2 | ENSG00000114423 | ENSG00000177885 | Q13191-1 | P62993-1 |
| CRK | ABL1 | ENSG00000167193 | ENSG00000097007 | P46108-1 | P00519-2 |
| CRK | ABL2 | ENSG00000167193 | ENSG00000143322 | P46108-1 | P42684-3 |
| CRK | ALS2CR12 | ENSG00000167193 | ENSG00000155749 | P46108-1 | Q96Q35-2 |
| CRK | ASAP1 | ENSG00000167193 | ENSG00000153317 | P46108-1 | Q9ULH1 |
| CRK | ASAP3 | ENSG00000167193 | ENSG00000088280 | P46108-1 | Q8TDY4-1 |
| CRK | ASB9 | ENSG00000167193 | ENSG00000102048 | P46108-1 | Q96DX5-1 |
| CRK | ATXN1 | ENSG00000167193 | ENSG00000124788 | P46108-1 | P54253-1 |
| CRK | BEX5 | ENSG00000167193 | ENSG00000184515 | P46108-1 | Q5H9J7 |
| CRK | BUB1 | ENSG00000167193 | ENSG00000169679 | P46108-1 | O43683-1 |
| CRK | CBL | ENSG00000167193 | ENSG00000110395 | P46108-1 | P22681 |
| CRK | CBLB | ENSG00000167193 | ENSG00000114423 | P46108-1 | Q13191-1 |
| CRK | CHTF18 | ENSG00000167193 | ENSG00000127586 | P46108-1 | Q8WVB6-1 |
| CRK | CNDP2 | ENSG00000167193 | ENSG00000133313 | P46108-1 | Q96KP4-1 |
| CRK | DOCK1 | ENSG00000167193 | ENSG00000150760 | P46108-1 | Q14185 |
| CRK | DOK2 | ENSG00000167193 | ENSG00000147443 | P46108-1 | O60496 |
| CRK | DOK3 | ENSG00000167193 | ENSG00000146094 | P46108-1 | Q7L591-3 |
| CRK | DOK7 | ENSG00000167193 | ENSG00000175920 | P46108-1 | Q18PE1-1 |
| CRK | ELK1 | ENSG00000167193 | ENSG00000126767 | P46108-1 | P19419-1 |
| CRK | ELK3 | ENSG00000167193 | ENSG00000111145 | P46108-1 | P41970 |
| CRK | EPS15 | ENSG00000167193 | ENSG00000085832 | P46108-1 | B1AUU8 |

| | | | | | |
|---|---|---|---|---|---|
| CRK | EYA3 | ENSG00000167193 | ENSG00000158161 | P46108-1 | Q99504-3 |
| CRK | FGFR1 | ENSG00000167193 | ENSG00000077782 | P46108-1 | P11362-2 |
| CRK | FLNA | ENSG00000167193 | ENSG00000196924 | P46108-1 | Q5HY54 |
| CRK | FSTL1 | ENSG00000167193 | ENSG00000163430 | P46108-1 | Q12841-1 |
| CRK | GAB1 | ENSG00000167193 | ENSG00000109458 | P46108-1 | Q13480-2 |
| CRK | GABPB2 | ENSG00000167193 | ENSG00000143458 | P46108-1 | Q8TAK5 |
| CRK | GRB2 | ENSG00000167193 | ENSG00000177885 | P46108-1 | P62993-1 |
| CRK | HSH2D | ENSG00000167193 | ENSG00000196684 | P46108-1 | Q96JZ2-1 |
| CRK | KCTD13 | ENSG00000167193 | ENSG00000174943 | P46108-1 | Q8WZ19 |
| CRK | KIT | ENSG00000167193 | ENSG00000157404 | P46108-1 | P10721 |
| CRK | LASP1 | ENSG00000167193 | ENSG00000002834 | P46108-1 | C9J9W2 |
| CRK | MAGEC3 | ENSG00000167193 | ENSG00000165509 | P46108-1 | Q8TD91-2 |
| CRK | MAP4K1 | ENSG00000167193 | ENSG00000104814 | P46108-1 | Q92918-1 |
| CRK | MAPK8 | ENSG00000167193 | ENSG00000107643 | P46108-1 | P45983-4 |
| CRK | MPG | ENSG00000167193 | ENSG00000103152 | P46108-1 | P29372-4 |
| CRK | MYLIP | ENSG00000167193 | ENSG00000007944 | P46108-1 | Q8WY64-1 |
| CRK | NUFIP2 | ENSG00000167193 | ENSG00000108256 | P46108-1 | Q7Z417-1 |
| CRK | PAFAH1B2 | ENSG00000167193 | ENSG00000168092 | P46108-1 | P68402-1 |
| CRK | PHC2 | ENSG00000167193 | ENSG00000134686 | P46108-1 | A0A0A0MSI2 |
| CRK | PIK3R1 | ENSG00000167193 | ENSG00000145675 | P46108-1 | P27986-2 |
| CRK | PIK3R2 | ENSG00000167193 | ENSG00000105647 | P46108-1 | E9PFP1 |
| CRK | PIK3R3 | ENSG00000167193 | ENSG00000117461 | P46108-1 | Q92569-1 |
| CRK | PLSCR1 | ENSG00000167193 | ENSG00000188313 | P46108-1 | O15162-1 |
| CRK | PPFIBP2 | ENSG00000167193 | ENSG00000166387 | P46108-1 | Q8ND30-1 |
| CRK | PRKACA | ENSG00000167193 | ENSG00000072062 | P46108-1 | P17612-1 |
| CRK | PSMC6 | ENSG00000167193 | ENSG00000100519 | P46108-1 | P62333 |
| CRK | PTK2 | ENSG00000167193 | ENSG00000169398 | P46108-1 | E9PEI4 |
| CRK | PTPN4 | ENSG00000167193 | ENSG00000088179 | P46108-1 | P29074 |
| CRK | RAB2B | ENSG00000167193 | ENSG00000129472 | P46108-1 | Q8WUD1-1 |
| CRK | RAPGEF1 | ENSG00000167193 | ENSG00000107263 | P46108-1 | Q13905-4 |
| CRK | RET | ENSG00000167193 | ENSG00000165731 | P46108-1 | P07949 |
| CRK | RIN3 | ENSG00000167193 | ENSG00000100599 | P46108-1 | A0A087WWY9 |
| CRK | RTCB | ENSG00000167193 | ENSG00000100220 | P46108-1 | Q9Y3I0 |
| CRK | SEMA4D | ENSG00000167193 | ENSG00000187764 | P46108-1 | Q92854-1 |
| CRK | SH2D2A | ENSG00000167193 | ENSG00000027869 | P46108-1 | Q9NP31-1 |
| CRK | SHC1 | ENSG00000167193 | ENSG00000160691 | P46108-1 | P29353-7 |
| CRK | SOCS1 | ENSG00000167193 | ENSG00000185338 | P46108-1 | O15524 |
| CRK | SOCS6 | ENSG00000167193 | ENSG00000170677 | P46108-1 | O14544 |
| CRK | SOS1 | ENSG00000167193 | ENSG00000115904 | P46108-1 | Q07889-1 |
| CRK | SOS2 | ENSG00000167193 | ENSG00000100485 | P46108-1 | Q07890-1 |
| CRK | SRC | ENSG00000167193 | ENSG00000197122 | P46108-1 | P12931-1 |
| CRK | STAT4 | ENSG00000167193 | ENSG00000138378 | P46108-1 | Q14765 |
| CRK | TCAP | ENSG00000167193 | ENSG00000173991 | P46108-1 | O15273 |
| CRK | TP53BP2 | ENSG00000167193 | ENSG00000143514 | P46108-1 | Q13625-2 |
| CRK | TXK | ENSG00000167193 | ENSG00000074966 | P46108-1 | P42681 |
| CRK | USP53 | ENSG00000167193 | ENSG00000145390 | P46108-1 | Q70EK8 |
| CRK | VAC14 | ENSG00000167193 | ENSG00000103043 | P46108-1 | Q08AM6-1 |
| DOCK1 | GRB2 | ENSG00000150760 | ENSG00000177885 | Q14185 | P62993-1 |
| DOK3 | DOK2 | ENSG00000146094 | ENSG00000147443 | Q7L591-3 | O60496 |

| | | | | | |
|---|---|---|---|---|---|
| EPS15 | ABL1 | ENSG00000085832 | ENSG00000097007 | B1AUU8 | P00519-2 |
| EPS15 | EPS15 | ENSG00000085832 | ENSG00000085832 | B1AUU8 | B1AUU8 |
| EPS15 | GRB2 | ENSG00000085832 | ENSG00000177885 | B1AUU8 | P62993-1 |
| FGFR1 | FGFR1 | ENSG00000077782 | ENSG00000077782 | P11362-2 | P11362-2 |
| FGFR1 | PIK3R1 | ENSG00000077782 | ENSG00000145675 | P11362-2 | P27986-2 |
| GAB1 | GRB2 | ENSG00000109458 | ENSG00000177885 | Q13480-2 | P62993-1 |
| GAB1 | PIK3R1 | ENSG00000109458 | ENSG00000145675 | Q13480-2 | P27986-2 |
| GAB1 | SHC1 | ENSG00000109458 | ENSG00000160691 | Q13480-2 | P29353-7 |
| GRB2 | FLNA | ENSG00000177885 | ENSG00000196924 | P62993-1 | Q5HY54 |
| GRB2 | GRB2 | ENSG00000177885 | ENSG00000177885 | P62993-1 | P62993-1 |
| GSPT1 | ETF1 | ENSG00000103342 | ENSG00000120705 | P15170-2 | P62495-2 |
| GSPT1 | PABPC1 | ENSG00000103342 | ENSG00000070756 | P15170-2 | P11940-1 |
| KCTD13 | KCTD13 | ENSG00000174943 | ENSG00000174943 | Q8WZ19 | Q8WZ19 |
| KIT | GRB2 | ENSG00000157404 | ENSG00000177885 | P10721 | P62993-1 |
| KIT | SOCS1 | ENSG00000157404 | ENSG00000185338 | P10721 | O15524 |
| KIT | SOCS6 | ENSG00000157404 | ENSG00000170677 | P10721 | O14544 |
| KPNA1 | ANP32B | ENSG00000114030 | ENSG00000136938 | P52294 | Q92688-1 |
| KPNA1 | CLK4 | ENSG00000114030 | ENSG00000113240 | P52294 | Q9HAZ1 |
| KPNA1 | KPNB1 | ENSG00000114030 | ENSG00000108424 | P52294 | Q14974-1 |
| KPNA1 | LMNB1 | ENSG00000114030 | ENSG00000113368 | P52294 | P20700 |
| KPNA1 | MACROD1 | ENSG00000114030 | ENSG00000133315 | P52294 | Q9BQ69 |
| KPNA1 | NMNAT1 | ENSG00000114030 | ENSG00000173614 | P52294 | Q9HAN9 |
| KPNA1 | NUP50 | ENSG00000114030 | ENSG00000093000 | P52294 | Q9UKX7-1 |
| KPNA1 | POLR3C | ENSG00000114030 | ENSG00000186141 | P52294 | Q9BUI4 |
| KPNA1 | RAG1 | ENSG00000114030 | ENSG00000166349 | P52294 | P15918 |
| KPNA1 | TAF9 | ENSG00000114030 | ENSG00000273841 | P52294 | Q16594 |
| KPNA1 | UBR5 | ENSG00000114030 | ENSG00000104517 | P52294 | O95071-1 |
| LASP1 | ATXN1 | ENSG00000002834 | ENSG00000124788 | C9J9W2 | P54253-1 |
| LASP1 | PLSCR1 | ENSG00000002834 | ENSG00000188313 | C9J9W2 | O15162-1 |
| LASP1 | SH2D2A | ENSG00000002834 | ENSG00000027869 | C9J9W2 | Q9NP31-1 |
| LASP1 | VAC14 | ENSG00000002834 | ENSG00000103043 | C9J9W2 | Q08AM6-1 |
| LMNB1 | TP53BP2 | ENSG00000113368 | ENSG00000143514 | P20700 | Q13625-2 |
| MAP4K1 | GRB2 | ENSG00000104814 | ENSG00000177885 | Q92918-1 | P62993-1 |
| MAPK8 | ELK1 | ENSG00000107643 | ENSG00000126767 | P45983-4 | P19419-1 |
| MECP2 | HIST2H3A | ENSG00000169057 | ENSG00000203852 | P51608-1 | Q71DI3 |
| MECP2 | HIST2H3C | ENSG00000169057 | ENSG00000203811 | P51608-1 | Q71DI3 |
| MECP2 | PRPF40A | ENSG00000169057 | ENSG00000196504 | P51608-1 | O75400-2 |
| MECP2 | SMARCA2 | ENSG00000169057 | ENSG00000080503 | P51608-1 | P51531-2 |
| MECP2 | YY1 | ENSG00000169057 | ENSG00000100811 | P51608-1 | P25490 |
| MELTF | MESD | ENSG00000163975 | ENSG00000117899 | P08582-2 | Q14696-1 |
| MELTF | NBPF19 | ENSG00000163975 | ENSG00000271383 | P08582-2 | NULL |
| MELTF | NOTCH2NL | ENSG00000163975 | ENSG00000264343 | P08582-2 | Q7Z3S9-1 |
| NMNAT1 | NMNAT1 | ENSG00000173614 | ENSG00000173614 | Q9HAN9 | Q9HAN9 |
| PABPC1 | PABPC1 | ENSG00000070756 | ENSG00000070756 | P11940-1 | P11940-1 |
| PHC2 | PHC2 | ENSG00000134686 | ENSG00000134686 | A0A0A0MSI2 | A0A0A0MSI2 |
| PIK3R1 | GRB2 | ENSG00000145675 | ENSG00000177885 | P27986-2 | P62993-1 |
| PIK3R1 | KIT | ENSG00000145675 | ENSG00000157404 | P27986-2 | P10721 |
| PIK3R1 | PTK2 | ENSG00000145675 | ENSG00000169398 | P27986-2 | E9PEI4 |
| PIK3R1 | SHC1 | ENSG00000145675 | ENSG00000160691 | P27986-2 | P29353-7 |

| | | | | | |
|---|---|---|---|---|---|
| PIK3R1 | SOCS1 | ENSG00000145675 | ENSG00000185338 | P27986-2 | O15524 |
| PIK3R1 | SRC | ENSG00000145675 | ENSG00000197122 | P27986-2 | P12931-1 |
| PIK3R2 | CBL | ENSG00000105647 | ENSG00000110395 | E9PFP1 | P22681 |
| PIK3R2 | GRB2 | ENSG00000105647 | ENSG00000177885 | E9PFP1 | P62993-1 |
| PIK3R2 | KIT | ENSG00000105647 | ENSG00000157404 | E9PFP1 | P10721 |
| PIK3R3 | ABL2 | ENSG00000117461 | ENSG00000143322 | Q92569-1 | P42684-3 |
| PIK3R3 | GRB2 | ENSG00000117461 | ENSG00000177885 | Q92569-1 | P62993-1 |
| PIK3R3 | HSH2D | ENSG00000117461 | ENSG00000196684 | Q92569-1 | Q96JZ2-1 |
| PIK3R3 | PTK2 | ENSG00000117461 | ENSG00000169398 | Q92569-1 | E9PEI4 |
| PIK3R3 | SOCS6 | ENSG00000117461 | ENSG00000170677 | Q92569-1 | O14544 |
| PIK3R3 | SRC | ENSG00000117461 | ENSG00000197122 | Q92569-1 | P12931-1 |
| PSMC6 | PSMC6 | ENSG00000100519 | ENSG00000100519 | P62333 | P62333 |
| PTK2 | GRB2 | ENSG00000169398 | ENSG00000177885 | E9PEI4 | P62993-1 |
| PTK2 | PTK2 | ENSG00000169398 | ENSG00000169398 | E9PEI4 | E9PEI4 |
| PTK2 | SRC | ENSG00000169398 | ENSG00000197122 | E9PEI4 | P12931-1 |
| RAPGEF1 | GRB2 | ENSG00000107263 | ENSG00000177885 | Q13905-4 | P62993-1 |
| SH2D2A | PIK3R1 | ENSG00000027869 | ENSG00000145675 | Q9NP31-1 | P27986-2 |
| SH2D2A | PIK3R3 | ENSG00000027869 | ENSG00000117461 | Q9NP31-1 | Q92569-1 |
| SH2D2A | PTK2 | ENSG00000027869 | ENSG00000169398 | Q9NP31-1 | E9PEI4 |
| SHC1 | GRB2 | ENSG00000160691 | ENSG00000177885 | P29353-7 | P62993-1 |
| SOS1 | GRB2 | ENSG00000115904 | ENSG00000177885 | Q07889-1 | P62993-1 |
| SOS1 | PIK3R1 | ENSG00000115904 | ENSG00000145675 | Q07889-1 | P27986-2 |
| SOS2 | GRB2 | ENSG00000100485 | ENSG00000177885 | Q07890-1 | P62993-1 |
| TP53BP2 | GRB2 | ENSG00000143514 | ENSG00000177885 | Q13625-2 | P62993-1 |
| TP53BP2 | TP53BP2 | ENSG00000143514 | ENSG00000143514 | Q13625-2 | Q13625-2 |
| TXK | DOK2 | ENSG00000074966 | ENSG00000147443 | P42681 | O60496 |
| TXK | GRB2 | ENSG00000074966 | ENSG00000177885 | P42681 | P62993-1 |
| TXK | PIK3R1 | ENSG00000074966 | ENSG00000145675 | P42681 | P27986-2 |
| TXK | SOCS6 | ENSG00000074966 | ENSG00000170677 | P42681 | O14544 |
| VAC14 | GABPB2 | ENSG00000103043 | ENSG00000143458 | Q08AM6-1 | Q8TAK5 |
| VAC14 | VAC14 | ENSG00000103043 | ENSG00000103043 | Q08AM6-1 | Q08AM6-1 |

**Table S5. List of protein-protein interactions generated using the Protein Interaction Network Analysis (PINA 3.0) platform, in the context of AML (The Cancer Genome Atlas, TCGA dataset).**

| Interactor A Protein ID | Interactor B Protein ID | Interactor A Gene ID | Interactor B Gene ID |
|---|---|---|---|
| P46108 | P00519 | CRK | ABL1 |
| P46108 | P60709 | CRK | ACTB |
| P46108 | Q13023 | CRK | AKAP6 |
| P46108 | Q96P48 | CRK | ARAP1 |
| P46108 | O15143 | CRK | ARPC1B |
| P46108 | Q9ULH1 | CRK | ASAP1 |
| P46108 | P54253 | CRK | ATXN1 |
| P46108 | P11274 | CRK | BCR |
| P46108 | P52907 | CRK | CAPZA1 |
| P46108 | P22681 | CRK | CBL |
| P46108 | Q9NPY3 | CRK | CD93 |
| P46108 | Q8WVB6 | CRK | CHTF18 |
| P46108 | Q9ULV4 | CRK | CORO1C |
| P46108 | Q8IU60 | CRK | DCP2 |

| | | | |
|---|---|---|---|
| P46108 | O60496 | CRK | DOK2 |
| P46108 | Q8TEW6 | CRK | DOK4 |
| P46108 | Q96F86 | CRK | EDC3 |
| P46108 | Q96JJ3 | CRK | ELMO2 |
| P46108 | P04626 | CRK | ERBB2 |
| P46108 | O43909 | CRK | EXTL3 |
| P46108 | P31994 | CRK | FCGR2B |
| P46108 | P31995 | CRK | FCGR2C |
| P46108 | Q96AE4 | CRK | FUBP1 |
| P46108 | Q96JZ2 | CRK | HSH2D |
| P46108 | P08238 | CRK | HSP90AB1 |
| P46108 | P17066 | CRK | HSPA6 |
| P46108 | Q9NZL4 | CRK | HSPBP1 |
| P46108 | Q96RY7 | CRK | IFT140 |
| P46108 | P10721 | CRK | KIT |
| P46108 | P02545 | CRK | LMNA |
| P46108 | P61626 | CRK | LYZ |
| P46108 | P41218 | CRK | MNDA |
| P46108 | Q14511 | CRK | NEDD9 |
| P46108 | Q14980 | CRK | NUMA1 |
| P46108 | O15162 | CRK | PLSCR1 |
| P46108 | P17612 | CRK | PRKACA |
| P46108 | Q9H4P4 | CRK | RNF41 |
| P46108 | P39019 | CRK | RPS19 |
| P46108 | P23396 | CRK | RPS3 |
| P46108 | P46782 | CRK | RPS5 |
| P46108 | P10301 | CRK | RRAS |
| P46108 | Q14141 | CRK | SEPTIN6 |
| P46108 | P08047 | CRK | SP1 |
| P46108 | P12931 | CRK | SRC |
| P46108 | Q99816 | CRK | TSG101 |
| P46108 | Q92558 | CRK | WASF1 |
| P46108 | P61981 | CRK | YWHAG |
| P15170 | P61221 | GSPT1 | ABCE1 |
| P15170 | P78371 | GSPT1 | CCT2 |
| P15170 | O14579 | GSPT1 | COPE |
| P15170 | Q66K64 | GSPT1 | DCAF15 |
| P15170 | Q16531 | GSPT1 | DDB1 |
| P15170 | O60231 | GSPT1 | DHX16 |
| P15170 | P23588 | GSPT1 | EIF4B |
| P15170 | Q15717 | GSPT1 | ELAVL1 |
| P15170 | P14735 | GSPT1 | IDE |
| P15170 | P01106 | GSPT1 | MYC |
| P15170 | P11940 | GSPT1 | PABPC1 |
| P15170 | Q9Y5S9 | GSPT1 | RBM8A |
| P15170 | P35241 | GSPT1 | RDX |
| P15170 | Q92541 | GSPT1 | RTF1 |
| P15170 | Q13573 | GSPT1 | SNW1 |
| P15170 | O95071 | GSPT1 | UBR5 |

| | | | |
|---|---|---|---|
| P15170 | P61758 | GSPT1 | VBP1 |
| P15170 | O14980 | GSPT1 | XPO1 |
| P52294 | Q9BTT0 | KPNA1 | ANP32E |
| P52294 | Q68CP9 | KPNA1 | ARID2 |
| P52294 | Q9NPI1 | KPNA1 | BRD7 |
| P52294 | Q86VP6 | KPNA1 | CAND1 |
| P52294 | P49427 | KPNA1 | CDC34 |
| P52294 | P55060 | KPNA1 | CSE1L |
| P52294 | P49711 | KPNA1 | CTCF |
| P52294 | Q13618 | KPNA1 | CUL3 |
| P52294 | Q13620 | KPNA1 | CUL4B |
| P52294 | Q58WW2 | KPNA1 | DCAF6 |
| P52294 | P29692 | KPNA1 | EEF1D |
| P52294 | P00533 | KPNA1 | EGFR |
| P52294 | P22087 | KPNA1 | FBL |
| P52294 | Q06546 | KPNA1 | GABPA |
| P52294 | Q86YP4 | KPNA1 | GATAD2A |
| P52294 | Q6FI13 | KPNA1 | H2AC18 |
| P52294 | Q9UBN7 | KPNA1 | HDAC6 |
| P52294 | Q16665 | KPNA1 | HIF1A |
| P52294 | Q8IYS2 | KPNA1 | KIAA2013 |
| P52294 | O00505 | KPNA1 | KPNA3 |
| P52294 | O95983 | KPNA1 | MBD3 |
| P52294 | Q00987 | KPNA1 | MDM2 |
| P52294 | Q7L2J0 | KPNA1 | MEPCE |
| P52294 | P46013 | KPNA1 | MKI67 |
| P52294 | Q9Y314 | KPNA1 | NOSIP |
| P52294 | P04150 | KPNA1 | NR3C1 |
| P52294 | P04629 | KPNA1 | NTRK1 |
| P52294 | P49790 | KPNA1 | NUP153 |
| P52294 | Q9UKX7 | KPNA1 | NUP50 |
| P52294 | P37198 | KPNA1 | NUP62 |
| P52294 | Q99623 | KPNA1 | PHB2 |
| P52294 | P62136 | KPNA1 | PPP1CA |
| P52294 | Q9UMS4 | KPNA1 | PRPF19 |
| P52294 | Q8WWY3 | KPNA1 | PRPF31 |
| P52294 | P49792 | KPNA1 | RANBP2 |
| P52294 | Q04206 | KPNA1 | RELA |
| P52294 | P39019 | KPNA1 | RPS19 |
| P52294 | Q9Y230 | KPNA1 | RUVBL2 |
| P52294 | Q9HC62 | KPNA1 | SENP2 |
| P52294 | Q15637 | KPNA1 | SF1 |
| P52294 | Q9HBD4 | KPNA1 | SMARCA4 |
| P52294 | P51532 | KPNA1 | SMARCA4 |
| P52294 | J3KMX2 | KPNA1 | SMARCD2 |
| P52294 | Q92925 | KPNA1 | SMARCD2 |
| P52294 | Q8N9Q2 | KPNA1 | SREK1IP1 |
| P52294 | P42224 | KPNA1 | STAT1 |
| P52294 | Q8WUJ0 | KPNA1 | STYX |

| | | | |
|---|---|---|---|
| P52294 | P11388 | KPNA1 | TOP2A |
| P52294 | Q14669 | KPNA1 | TRIP12 |
| P52294 | Q96FX7 | KPNA1 | TRMT61A |
| P52294 | Q9Y5U2 | KPNA1 | TSSC4 |
| P52294 | Q71U36 | KPNA1 | TUBA1A |
| P52294 | P61081 | KPNA1 | UBE2M |
| P52294 | O94782 | KPNA1 | USP1 |
| P52294 | Q93009 | KPNA1 | USP7 |
| P52294 | Q15906 | KPNA1 | VPS72 |
| P51608 | Q9Y587 | MECP2 | AP4S1 |
| P51608 | Q9NRL2 | MECP2 | BAZ1A |
| P51608 | Q13185 | MECP2 | CBX3 |
| P51608 | P45973 | MECP2 | CBX5 |
| P51608 | P60953 | MECP2 | CDC42 |
| P51608 | Q9H444 | MECP2 | CHMP4B |
| P51608 | Q9H8H2 | MECP2 | DDX31 |
| P51608 | Q8WYQ5 | MECP2 | DGCR8 |
| P51608 | Q9UBC3 | MECP2 | DNMT3B |
| P51608 | P98174 | MECP2 | FGD1 |
| P51608 | O00358 | MECP2 | FOXE1 |
| P51608 | O15379 | MECP2 | HDAC3 |
| P51608 | Q96PV6 | MECP2 | LENG8 |
| P51608 | Q06330 | MECP2 | RBPJ |
| P51608 | Q9UNX3 | MECP2 | RPL26L1 |
| P51608 | Q14684 | MECP2 | RRP1B |
| P51608 | Q96ST3 | MECP2 | SIN3A |
| P51608 | P17947 | MECP2 | SPI1 |
| P51608 | P61088 | MECP2 | UBE2N |
| P51608 | O60287 | MECP2 | URB1 |
| P51608 | P12956 | MECP2 | XRCC6 |
| P51608 | P67809 | MECP2 | YBX1 |
| P08582 | Q96Q80 | MELTF | DERL3 |

**Table S6. miRNA-Disease analysis outcome (from miRNet).**

| Disease | Group | Hits | Pval | FDR |
|---|---|---|---|---|
| Crohn Disease | Autoimmune Disorders | 9 | $1.39X10^{-4}$ | $6.04X10^{-4}$ |
| Pulmonary Sarcoidosis | Autoimmune Disorders | 4 | $4.82X10^{-3}$ | $1.00X10^{-2}$ |
| Myasthenia Gravis | Autoimmune Disorders | 4 | $1.15X10^{-2}$ | $1.79X10^{-2}$ |
| Psoriasis | Autoimmune Disorders | 6 | $1.50X10^{-2}$ | $2.08X10^{-2}$ |
| Systemic Lupus Erythematosus | Autoimmune Disorders | 8 | $1.79X10^{-2}$ | $2.39X10^{-2}$ |
| Carcinoma Renal Cell | Cancers: Carcinomas | 15 | $1.37X10^{-6}$ | $4.57X10^{-5}$ |
| Squamous Cell Carcinoma Head and Neck | Cancers: Carcinomas | 12 | $1.32X10^{-5}$ | $1.20X10^{-4}$ |
| Carcinoma Lung Non-Small-Cell | Cancers: Carcinomas | 13 | $3.07X10^{-5}$ | $2.05X10^{-4}$ |
| Adenocarcinoma Gastric | Cancers: Carcinomas | 10 | $5.07X10^{-5}$ | $2.81X10^{-4}$ |
| Carcinoma Nasopharyngeal | Cancers: Carcinomas | 12 | $1.62X10^{-4}$ | $6.75X10^{-4}$ |
| Carcinoma Endometrial | Cancers: Carcinomas | 11 | $4.02X10^{-4}$ | $1.61X10^{-3}$ |
| Carcinoma Breast Triple Negative | Cancers: Carcinomas | 11 | $4.98X10^{-4}$ | $1.84X10^{-3}$ |
| Carcinoma Ovarian Serous | Cancers: Carcinomas | 6 | $3.20X10^{-3}$ | $7.62X10^{-3}$ |
| Carcinoma Cervical | Cancers: Carcinomas | 10 | $3.43X10^{-3}$ | $7.62X10^{-3}$ |

| | | | | |
|---|---|---|---|---|
| Carcinoma Esophageal | Cancers: Carcinomas | 10 | 3.43X10$^{-3}$ | 7.62X10$^{-3}$ |
| Carcinoma Gallbladder | Cancers: Carcinomas | 6 | 5.88X10$^{-3}$ | 1.18X10$^{-2}$ |
| Carcinoma Breast | Cancers: Carcinomas | 9 | 7.84X10$^{-3}$ | 1.45X10$^{-2}$ |
| Carcinoma Laryngeal | Cancers: Carcinomas | 9 | 7.84X10$^{-3}$ | 1.45X10$^{-2}$ |
| Choriocarcinoma | Cancers: Carcinomas | 9 | 8.79X10$^{-3}$ | 1.59X10$^{-2}$ |
| Carcinoma Adrenocortical | Cancers: Carcinomas | 6 | 8.91X10$^{-3}$ | 1.59X10$^{-2}$ |
| Hereditary Breast Carcinoma | Cancers: Carcinomas | 2 | 1.17X10$^{-2}$ | 1.79X10$^{-2}$ |
| Carcinoma Colon | Cancers: Carcinomas | 9 | 1.18X10$^{-2}$ | 1.79X10$^{-2}$ |
| Early-Stage Gastric Carcinoma | Cancers: Carcinomas | 3 | 1.71X10$^{-2}$ | 2.34X10$^{-2}$ |
| Carcinoma Renal Cell Clear-Cell | Cancers: Carcinomas | 8 | 2.19X10$^{-2}$ | 2.81X10$^{-2}$ |
| Carcinoma Hepatocellular HBV-Related | Cancers: Carcinomas | 4 | 2.38X10$^{-2}$ | 2.98X10$^{-2}$ |
| Carcinoma Bladder | Cancers: Carcinomas | 8 | 2.66X10$^{-2}$ | 3.02X10$^{-2}$ |
| Adenocarcinoma Pancreatic Ductal | Cancers: Carcinomas | 8 | 2.84X10$^{-2}$ | 3.16X10$^{-2}$ |
| Carcinoma Pancreatic | Cancers: Carcinomas | 8 | 2.93X10$^{-2}$ | 3.18X10$^{-2}$ |
| Squamous Cell Carcinoma Esophageal | Cancers: Carcinomas | 8 | 2.93X10$^{-2}$ | 3.18X10$^{-2}$ |
| Carcinoma Urothelial Upper Tract | Cancers: Carcinomas | 5 | 3.04X10$^{-2}$ | 3.23X10$^{-2}$ |
| Glioblastoma | Cancers: Gliomas | 11 | 7.13X10$^{-4}$ | 2.55X10$^{-3}$ |
| Astrocytoma | Cancers: Gliomas | 5 | 3.11X10$^{-3}$ | 7.62X10$^{-3}$ |
| Glioma | Cancers: Gliomas | 10 | 3.29X10$^{-3}$ | 7.62X10$^{-3}$ |
| Leukemia Myeloid Chronic | Cancers: Leukemias | 13 | 1.82X10$^{-5}$ | 1.40X10$^{-4}$ |
| Leukemia Lymphoblastic Acute | Cancers: Leukemias | 6 | 3.20X10$^{-3}$ | 7.62X10$^{-3}$ |
| Leukemia | Cancers: Leukemias | 9 | 1.02X10$^{-2}$ | 1.67X10$^{-2}$ |
| Leukemia Myeloid Acute | Cancers: Leukemias | 9 | 1.31X10$^{-2}$ | 1.90X10$^{-2}$ |
| Lymphoma Primary Effusion | Cancers: Lymphomas | 8 | 8.27X10$^{-7}$ | 4.14X10$^{-5}$ |
| Lymphoma Mantle-Cell | Cancers: Lymphomas | 4 | 4.55X10$^{-4}$ | 1.75X10$^{-3}$ |
| Cutaneous Melanoma | Cancers: Melanomas | 7 | 1.32X10$^{-4}$ | 6.00X10$^{-4}$ |
| Melanoma | Cancers: Melanomas | 11 | 8.27X10$^{-4}$ | 2.76X10$^{-3}$ |
| Pancreatic Neoplasms | Cancers: Neoplasms | 17 | 3.82X10$^{-8}$ | 3.82X10$^{-6}$ |
| Pituitary Neoplasms | Cancers: Neoplasms | 8 | 3.73X10$^{-6}$ | 6.22X10$^{-5}$ |
| Colon Neoplasms | Cancers: Neoplasms | 14 | 7.74X10$^{-6}$ | 9.52X10$^{-5}$ |
| Lung Neoplasms | Cancers: Neoplasms | 14 | 8.86X10$^{-6}$ | 9.52X10$^{-5}$ |
| Head And Neck Neoplasms | Cancers: Neoplasms | 10 | 5.07X10$^{-5}$ | 2.81X10$^{-4}$ |
| Retinoblastoma | Cancers: Neoplasms | 11 | 6.22X10$^{-5}$ | 3.11X10$^{-4}$ |
| Malignant Neoplasms [unspecific] | Cancers: Neoplasms | 11 | 1.31X10$^{-4}$ | 6.00X10$^{-4}$ |
| Ovarian Neoplasms | Cancers: Neoplasms | 10 | 3.43X10$^{-3}$ | 7.62X10$^{-3}$ |
| Colon Adenoma | Cancers: Neoplasms | 5 | 5.22X10$^{-3}$ | 1.07X10$^{-2}$ |
| Gastrointestinal Neoplasms | Cancers: Neoplasms | 9 | 9.48X10$^{-3}$ | 1.62X10$^{-2}$ |
| Breast Neoplasms | Cancers: Neoplasms | 8 | 2.84X10$^{-2}$ | 3.16X10$^{-2}$ |
| Neoplasms [unspecific] | Cancers: Neoplasms | 8 | 3.20X10$^{-2}$ | 3.27X10$^{-2}$ |
| Osteosarcoma | Cancers: Sarcomas | 11 | 7.49X10$^{-4}$ | 2.58X10$^{-3}$ |
| Kaposi's Sarcoma | Cancers: Sarcomas | 7 | 1.15X10$^{-3}$ | 3.59X10$^{-3}$ |
| Soft Tissue Sarcoma | Cancers: Sarcomas | 3 | 1.08X10$^{-2}$ | 1.74X10$^{-2}$ |
| Atypical Teratoid Tumor | Cancers: Tumors | 2 | 6.44X10$^{-3}$ | 1.24X10$^{-2}$ |
| Neuroblastoma | Cancers: Tumors | 8 | 2.05X10$^{-2}$ | 2.70X10$^{-2}$ |
| ACTH-Secreting Pituitary Adenoma | Cancers: Tumors | 3 | 3.24X10$^{-2}$ | 3.27X10$^{-2}$ |
| Stroke Ischemic | Cardiovascular Diseases | 11 | 3.11X10$^{-6}$ | 6.22X10$^{-5}$ |
| Vascular Hypertrophy | Cardiovascular Diseases | 8 | 2.81X10$^{-5}$ | 2.01X10$^{-4}$ |
| Pulmonary Hypertension | Cardiovascular Diseases | 10 | 4.67X10$^{-5}$ | 2.81X10$^{-4}$ |
| Myocardial Infarction | Cardiovascular Diseases | 9 | 2.28X10$^{-3}$ | 6.82X10$^{-3}$ |

| | | | | |
|---|---|---|---|---|
| Heart Failure | Cardiovascular Diseases | 10 | 3.15X10-3 | 7.62X10-3 |
| Peripheral Vascular Disease | Cardiovascular Diseases | 4 | 1.15X10-2 | 1.79X10-2 |
| Coronary Heart Diseases | Cardiovascular Diseases | 8 | 1.79X10-2 | 2.39X10-2 |
| Cerebral Ischemia | Cardiovascular Diseases | 3 | 3.24X10-2 | 3.27X10-2 |
| Atherosclerosis | Cardiovascular Diseases | 8 | 3.40X10-2 | 3.40X10-2 |
| Macular Degeneration | Eye Disorders | 4 | 9.65X10-3 | 1.62X10-2 |
| Pain [unspecific] | General Disorders | 2 | 2.61X10-2 | 3.02X10-2 |
| Hemoglobin Diseases | Hematological Disorders | 7 | 9.52X10-6 | 9.52X10-5 |
| Myelodysplastic Syndromes | Hematological Disorders | 5 | 2.27X10-2 | 2.87X10-2 |
| Human Immunodeficiency Virus Infection | Infectious Diseases | 12 | 1.54X10-5 | 1.28X10-4 |
| Hepatitis C Virus Infection | Infectious Diseases | 13 | 5.34X10-5 | 2.81X10-4 |
| Lupus Vulgaris | Infectious Diseases | 3 | 3.53X10-3 | 7.67X10-3 |
| Sepsis | Infectious Diseases | 6 | 1.50X10-2 | 2.08X10-2 |
| Tuberculosis Pulmonary | Infectious Diseases | 5 | 2.16X10-2 | 2.81X10-2 |
| Epstein-Barr Virus Infection | Infectious Diseases | 4 | 2.53X10-2 | 3.02X10-2 |
| Chronic Kidney Disease | Kidney Disorders | 7 | 2.82X10-3 | 7.62X10-3 |
| Kidney Injury | Kidney Disorders | 5 | 3.18X10-2 | 3.27X10-2 |
| Liver Diseases [unspecific] | Liver Disorders | 10 | 5.64X10-6 | 8.06X10-5 |
| Alcoholic Hepatitis | Liver Disorders | 4 | 2.98X10-3 | 7.62X10-3 |
| Chronic Hepatitis B | Liver Disorders | 6 | 1.29X10-2 | 1.90X10-2 |
| Liver Injury | Liver Disorders | 4 | 1.46X10-2 | 2.08X10-2 |
| Fatty Liver [unspecific] | Liver Disorders | 2 | 2.61X10-2 | 3.02X10-2 |
| Gallstones | Liver Disorders | 2 | 2.61X10-2 | 3.02X10-2 |
| Diabetic Retinopathy | Metabolic Disorders | 9 | 2.18X10-6 | 5.45X10-5 |
| Diabetes Mellitus | Metabolic Disorders | 11 | 8.68X10-4 | 2.80X10-3 |
| Diabetes Mellitus Type 2 | Metabolic Disorders | 10 | 2.32X10-3 | 6.82X10-3 |
| Obesity | Metabolic Disorders | 8 | 9.72X10-3 | 1.62X10-2 |
| Diabetic Vasculopathy | Metabolic Disorders | 2 | 2.61X10-2 | 3.02X10-2 |
| Alzheimer's Disease | Neurological Disorders | 10 | 2.77X10-3 | 7.62X10-3 |
| Hyperactivity Disorder | Neurological Disorders | 2 | 2.61X10-2 | 3.02X10-2 |
| Epilepsy | Neurological Disorders | 5 | 2.64X10-2 | 3.02X10-2 |
| Non-Traumatic Subarachnoid Hemorrhage | Neurological Disorders | 3 | 3.24X10-2 | 3.27X10-2 |
| Muscular Dystrophy | Neuromuscular Disorders | 6 | 6.26X10-3 | 1.23X10-2 |
| Preeclampsia | Pregnancy-Related Disorders | 10 | 4.04X10-3 | 8.60X10-3 |
| Lung Injury [unspecific] | Respiratory Disorders | 3 | 9.43X10-3 | 1.62X10-2 |
| Asthma | Respiratory Disorders | 7 | 1.23X10-2 | 1.84X10-2 |
| Chronic Obstructive Pulmonary Disease | Respiratory Disorders | 5 | 3.04X10-2 | 3.23X10-2 |

*In red: Leukemia Myeloid Acute (AML).

**Table S7. List of Gene Expression Omnibus (GEO) databases analyzed in the study involving workers exposed to benzene.**

| GEO ID | Contributors | Platform | Samples (*H. sapiens*) | Reference |
|---|---|---|---|---|
| GSE21862 | Hubbard A et al. | Illumina HumanRef-8 v2.0 Expression BeadChip | 144 (96=E; 48=NE) | [1] |
| GSE9569 | McHale CM et al. | Affymetrix Human U133 GeneChip | 8 (4=E; 4=NE) | [2] |
| GSE50967 | Gao A et al. | Illumina Human Methylation 450 BeadChip | 12 (8=E; 4=NE) | [3] |

E, exposed to benzene; NE, not exposed to benzene.

**Table S8. List of Gene Expression Omnibus (GEO) databases analyzed in the study involving acute myeloid leukemia patients.**

| GEO ID | Contributors | Platform | Samples (H. sapiens) | Specimen | Reference |
|---|---|---|---|---|---|
| GSE171654 | Hanoun M et al. | Affymetrix Clariom S Human Transcriptome Array | 11 (AML=8; H=3) | BM-MSCs | [4] |

| | | | | | |
|---|---|---|---|---|---|
| GSE114868 | Huang H et al. | Affymetrix Human Transcriptome Array 2.0 | 214 (AML=194; H=20) | BM-MSCs | [5] |
| GSE92778 | Boyd A et al. | Affymetrix Whole-Transcript Human Gene 1.0 ST Array | 12 (AML=6; H=6) | BM-MSCs | [6] |
| GSE90062 | Li K et al. | Affymetrix Human Gene Expression Array | 6 (AML=3; H=3) | BM-HSCc | [7] |
| GSE84881 | von der Heide EK et al. | Affymetrix Human Genome Expression U133 Plus 2.0 Array | 23 (AML=19; H=4) | BM-MSCs | [8] |
| GSE79605 | Zheng X et al. | Agilent Human Gene Expression 4x44K v2 Microarray | 4 (AML=2; H=2) | BM-MSCs | n.a. |
| GSE65409 | Tan SF et al. | Illumina Human HT-12 V3.0 Expression BeadChip | 38 (AML=30; H=8) | PBMCs | [9] |
| GSE14924 | Le Dieu R et al. | Affymetrix Human Genome Expression U133 Plus 2.0 Array | 41 (AML=20; H=21) | PBTCs | [10] |
| GSE97485 | Hourigan CS et al. | Affymetrix Whole-Transcript Human Gene 1.0 ST Array | 20 (AML=10; H=10) | PBMCs | [11] |
| GSE109179 | Knaus H et al. | Affymetrix Human Gene Expression Array | 19 (AML=15; H=4) | PBTCs | [12] |

H, healthy subjects; AML, acute myeloid leukemia affected subjects; BM, bone marrow; MSCs, mesenchymal stem cells; HSCs, hematopoietic stem cells; PBMCs, peripheral blood myeloid cells; PBTCs, peripheral blood T cells; n.a., not associated.

## References


1. McHale, C.M.; Zhang, L.; Lan, Q.; Vermeulen, R.; Li, G.; Hubbard, A.E.; Porter, K.E.; Thomas, R.; Portier, C.J.; Shen, M.; et al. Global Gene Expression Profiling of a Population Exposed to a Range of Benzene Levels. *Environ. Health Perspect.* **2011**, *119*, 628–640, doi:10.1289/ehp.1002546.
2. McHale, C.M.; Zhang, L.; Lan, Q.; Li, G.; Hubbard, A.E.; Forrest, M.S.; Vermeulen, R.; Chen, J.; Shen, M.; Rappaport, S.M.; et al. Changes in the Peripheral Blood Transcriptome Associated with Occupational Benzene Exposure Identified by Cross-Comparison on Two Microarray Platforms. *Genomics* **2009**, *93*, 343–349, doi:10.1016/j.ygeno.2008.12.006.
3. Bai, W.; Yang, J.; Yang, G.; Niu, P.; Tian, L.; Gao, A. Long Non-Coding RNA NR_045623 and NR_028291 Involved in Benzene Hematotoxicity in Occupationally Benzene-Exposed Workers. *Exp. Mol. Pathol.* **2014**, *96*, 354–360, doi:10.1016/j.yexmp.2014.02.016.
4. Chen, Y.; Hoffmeister, L.M.; Zaun, Y.; Arnold, L.; Schmid, K.W.; Giebel, B.; Klein-Hitpass, L.; Hanenberg, H.; Squire, A.; Reinhardt, H.C.; et al. Acute Myeloid Leukemia–Induced Remodeling of the Human Bone Marrow Niche Predicts Clinical Outcome. *Blood Adv.* **2020**, *4*, 5257–5268, doi:10.1182/bloodadvances.2020001808.
5. Huang, H.-H.; Chen, F.-Y.; Chou, W.-C.; Hou, H.-A.; Ko, B.-S.; Lin, C.-T.; Tang, J.-L.; Li, C.-C.; Yao, M.; Tsay, W.; et al. Long Non-Coding RNA HOXB-AS3 Promotes Myeloid Cell Proliferation and Its Higher Expression Is an Adverse Prognostic Marker in Patients with Acute Myeloid Leukemia and Myelodysplastic Syndrome. *BMC Cancer* **2019**, *19*, 617, doi:10.1186/s12885-019-5822-y.
6. Boyd, A.L.; Reid, J.C.; Salci, K.R.; Aslostovar, L.; Benoit, Y.D.; Shapovalova, Z.; Nakanishi, M.; Porras, D.P.; Almakadi, M.; Campbell, C.J. V.; et al. Acute Myeloid Leukaemia Disrupts Endogenous Myelo-Erythropoiesis by Compromising the Adipocyte Bone Marrow Niche. *Nat. Cell Biol.* **2017**, *19*, 1336–1347, doi:10.1038/ncb3625.
7. Li, K.; Wang, F.; Cao, W.-B.; Lv, X.-X.; Hua, F.; Cui, B.; Yu, J.-J.; Zhang, X.-W.; Shang, S.; Liu, S.-S.; et al. TRIB3 Promotes APL Progression through Stabilization of the Oncoprotein PML-RARα and Inhibition of P53-Mediated Senescence. *Cancer Cell* **2017**, *31*, 697-710.e7, doi:10.1016/j.ccell.2017.04.006.
8. von der Heide, E.K.; Neumann, M.; Vosberg, S.; James, A.R.; Schroeder, M.P.; Ortiz-Tanchez, J.; Isaakidis, K.; Schlee, C.; Luther, M.; Jöhrens, K.; et al. Molecular Alterations in Bone Marrow Mesenchymal Stromal Cells Derived from Acute Myeloid Leukemia Patients. *Leukemia* **2017**, *31*, 1069–1078, doi:10.1038/leu.2016.324.
9. Tan, S.-F.; Liu, X.; Fox, T.E.; Barth, B.M.; Sharma, A.; Turner, S.D.; Awwad, A.; Dewey, A.; Doi, K.; Spitzer, B.; et al. Acid Ceramidase Is Upregulated in AML and Represents a Novel Therapeutic Target. *Oncotarget* **2016**, *7*, 83208–83222, doi:10.18632/oncotarget.13079.
10. Le Dieu, R.; Taussig, D.C.; Ramsay, A.G.; Mitter, R.; Miraki-Moud, F.; Fatah, R.; Lee, A.M.; Lister, T.A.; Gribben, J.G. Peripheral Blood T Cells in Acute Myeloid Leukemia (AML) Patients at Diagnosis Have Abnormal Phenotype and Genotype and Form Defective Immune Synapses with AML Blasts. *Blood* **2009**, *114*, 3909–3916, doi:10.1182/blood-2009-02-206946.
11. Goswami, M.; Prince, G.; Biancotto, A.; Moir, S.; Kardava, L.; Santich, B.H.; Cheung, F.; Kotliarov, Y.; Chen, J.; Shi, R.; et al. Impaired B Cell Immunity in Acute Myeloid Leukemia Patients after Chemotherapy. *J. Transl. Med.* **2017**, *15*, 155, doi:10.1186/s12967-017-1252-2.
12. Knaus, H.A.; Berglund, S.; Hackl, H.; Blackford, A.L.; Zeidner, J.F.; Montiel-Esparza, R.; Mukhopadhyay, R.; Vanura, K.; Blazar, B.R.; Karp, J.E.; et al. Signatures of CD8+ T Cell Dysfunction in AML Patients and Their Reversibility with Response to Chemotherapy. *JCI Insight* **2018**, *3*, doi:10.1172/jci.insight.120974.
